# Multi-Evidence Clinical Reasoning With Retrieval-Augmented Generation for Emergency Triage: Retrospective Evaluation Study

**DOI:** 10.2196/82026

**Published:** 2026-01-26

**Authors:** Hang Sheung Wong, Tsz Kwan Wong

**Affiliations:** 1 Department of Accident & Emergency Princess Margaret Hospital Hong Kong (SAR) China (Hong Kong); 2 Department of Accident & Emergency North Lantau Hospital Hong Kong (SAR) China (Hong Kong)

**Keywords:** artificial intelligence, clinical decision support, clinical informatics, DeepSeek, digital health, emergency medicine, large language models, nurses, retrieval-augmented generation, triage

## Abstract

**Background:**

Emergency triage accuracy is critical but varies with clinician experience, cognitive load, and case complexity. Mis-triage can delay care for high-risk patients and exacerbate crowding through unnecessary prioritization. Large language models (LLMs) show promise as triage decision-support tools but are vulnerable to hallucinations. Retrieval-augmented generation (RAG) may improve reliability by grounding LLM reasoning in authoritative guidelines and real clinical cases.

**Objective:**

This study aimed to evaluate whether a dual-source RAG system that integrates guideline- and case-based evidence improves emergency triage performance versus a baseline LLM and to assess how closely its urgency assignments align with expert consensus and outcome-defined clinical severity.

**Methods:**

We developed a dual-source RAG system—Multi-Evidence Clinical Reasoning RAG (MECR-RAG)—that retrieves sections from the Hong Kong Accident and Emergency Triage Guidelines (HKAETG) and cases from a database of 3000 emergency department triage encounters. In a retrospective single‑center evaluation, MECR‑RAG and a prompt‑only baseline LLM (both DeepSeek‑V3) were tested on 236 routine triage encounters to predict 5‑level triage categories. Expert consensus reference labels were assigned by blinded senior triage nurses. Primary outcomes were quadratic weighted kappa (QWK) and accuracy versus consensus labels. Secondary analyses examined performance within 3 operationally and clinically relevant triage bands—immediate (categories 1 and 2), urgent (category 3), and nonurgent (categories 4 and 5). In 226 encounters with follow‑up, we also assigned outcome‑based severity tiers (R1-R3) using a published 3‑level urgency reference standard and defined a disposition‑safety composite.

**Results:**

MECR‑RAG achieved a mean QWK of 0.902 (SD 0.0021; 95% CI 0.901-0.904) and accuracy of 0.802 (SD 0.0082; 95% CI 0.795-0.808), outperforming the baseline LLM (QWK 0.801, SD 0.004; accuracy 0.542, SD 0.0073; both *P*<.001) and demonstrating expert‑comparable agreement with triage nurses (interrater QWK 0.887). In 3‑group analysis, MECR‑RAG reduced overtriage from 68/236 (28.8%) with the baseline LLM to 30/236 (12.7%) and maintained low undertriage from 4/236 (1.7%) to 3/236 (1.3%), with the largest gains in the diagnostically ambiguous yet operationally important categories 3 and 4. In a secondary outcome‑based analysis defining high‑severity courses as R1+R2, MECR‑RAG detected high-risk patients more sensitively than initial nurse triage (124/130, 95.4% vs 117/130, 90.0%; *P*=.02) while maintaining nurse‑level specificity. MECR‑RAG yielded the lowest weighted harm index (13.7, 19.5, and 20.3 per 100 patients for MECR‑RAG, nurses, and the baseline LLM, respectively).

**Conclusions:**

A dual‑source RAG triage system that combines guideline‑based rules with case‑based reasoning achieved expert‑comparable agreement, reduced overtriage, and better aligned urgency assignments than a prompt‑only baseline LLM. Secondary outcome–based analyses in this cohort suggested more favorable triage patterns than initial nurse triage, supporting MECR‑RAG as a concurrent decision‑support layer that flags discordant or high‑risk assignments; prospective multicenter implementation studies are needed to determine effects on emergency department crowding, delays, and patient outcomes.

## Introduction

Emergency triage is a critical step in emergency department (ED) operations, determining which patients are seen first when demand exceeds capacity. However, triage accuracy varies with clinicians’ experience, cognitive load, and the complexity of presenting symptoms, and even trained nurses may misclassify a substantial minority of encounters [1,2]. Undertriage risks delayed recognition of life-threatening illness and preventable morbidity or mortality, whereas overtriage diverts scarce resources toward lower-acuity patients and can exacerbate ED crowding and boarding, both of which are associated with worse outcomes [2]. These challenges are particularly pronounced in mid‑acuity categories, which represent most ED visits and where small changes in urgency assignment can translate into large differences in waiting time, crowding pressure, and exposure to harm.

Large language models (LLMs) have emerged as potential tools for clinical decision support because they can interpret free-text notes, integrate medical knowledge, and generate structured recommendations. In emergency care, LLMs have been tested for triage on simulated vignettes and curated cases, but reported performance has been highly variable, with agreement with human raters (Cohen κ) ranging from 0.125 to 0.899 across models, datasets, and evaluation frameworks [3-8]. A fundamental limitation of current “prompt-only” LLMs is their tendency to hallucinate, generating outputs that appear plausible but are factually incorrect or unsupported [9]. In emergency triage, such hallucinations may lead to urgency levels being assigned based on fabricated reasoning, outdated rules, or misinterpretation of presenting complaints, posing potential risks to patient safety and consistency.

Retrieval-augmented generation (RAG) has been proposed as a way to mitigate hallucinations by grounding LLM outputs in external, task-specific knowledge sources such as clinical guidelines, textbooks, electronic health records, and biomedical literature [10-12]. By retrieving relevant evidence at inference time and conditioning generation on that material, RAG can improve access to current, domain-specific knowledge and make model reasoning more transparent. Early work in emergency care has shown that adding retrieval can improve triage or referral accuracy compared with nonretrieval models. Yazaki et al [13] integrated RAG with a triage guideline database and tested GPT‑3.5 on 100 structured, simulated scenarios derived from the Japanese National Examination for Emergency Medical Technicians, finding that the RAG-enhanced model achieved 70/100 (70%) accuracy and outperformed both emergency medical technicians and physicians. Gaber et al [14] used a curated dataset of 2000 real-world ED cases from the MIMIC‑IV (Medical Information Mart for Intensive Care IV) database and retrieved evidence from a corpus of 30,000 PubMed abstracts, demonstrating superior triage-level prediction and exact-match accuracy compared with nonretrieval workflows.

Despite these advances, existing applications of RAG to emergency triage and referral have important gaps. One study relied largely on textbook-style, simulated examination cases [13], whereas another used routine labels from structured research datasets without clinician-adjudicated ground truth [14]. In both studies, RAG was applied to a single knowledge source—either structured triage guidelines or biomedical literature—rather than explicitly combining protocol‑based rules with experiential knowledge from prior cases.

To our knowledge, no prior work has evaluated a dual‑source RAG system that jointly retrieves local triage guidelines and real‑world past triage cases, or directly compared such a system with a prompt‑only LLM using raw, unstructured triage documentation paired with a blinded expert consensus reference standard and outcome‑based validity checks. This design better captures the complexity and variability of real-world practice and strengthens the translational relevance of performance estimates.

There is also growing recognition that agreement with human labels alone is an incomplete measure of triage quality. Large cohort studies of systems such as the Emergency Severity Index (ESI) and the Manchester Triage System (MTS) have shown that nurse-assigned categories can underestimate or overestimate “true” acuity when judged against downstream outcomes such as hospitalization, intensive care unit (ICU) transfer, revisits, and short-term mortality [15-17]. In parallel, a *Journal of the American Medical Association* (*JAMA*) editorial on artificial intelligence (AI) in medicine has argued that evaluations of AI tools should prioritize clinically meaningful outcomes, patient-centered care, quality, and equity rather than focusing solely on narrow technical accuracy metrics [18]. Accordingly, evaluations of LLM‑enabled triage should explicitly examine how assigned urgency levels align with downstream clinical severity and sentinel safety events, rather than treating concordance with nurse labels as sufficient.

In this context, we developed a dual-source RAG system—Multi-Evidence Clinical Reasoning RAG (MECR-RAG)—that retrieves both structured local triage guidelines (Hong Kong Accident and Emergency Triage Guidelines [HKAETG]) and analogous past triage cases and applies a structured Multi-Evidence Clinical Reasoning (MECR) framework to assign 5-level urgency categories. We retrospectively evaluated MECR-RAG on real ED triage notes, benchmarking it against a prompt-only baseline LLM and against expert nurse consensus labels. Our primary aim was to determine whether a dual‑source RAG triage system (MECR‑RAG) improves 5‑level triage accuracy and agreement (quadratic weighted κ and accuracy) with expert nurse consensus compared with a prompt‑only baseline LLM and to benchmark its performance against expert triage nurses. We hypothesized that MECR‑RAG would achieve higher agreement than the baseline LLM and noninferior agreement relative to expert nurses. As a secondary aim, in a subset with follow‑up data, we assessed outcome‑informed validity by examining how MECR‑RAG, the baseline LLM, and initial nurse triage aligned with downstream clinical severity tiers and a composite of sentinel safety outcomes. This early‑phase, single‑center evaluation, reported in accordance with TRIPOD‑LLM (Transparent Reporting of a multivariable prediction model for Individual Prognosis Or Diagnosis - Large Language Models) [19], is intended to provide a methodologically rigorous foundation for future prospective and implementation studies of RAG‑enhanced triage decision-support systems designed to augment, rather than replace, nurse‑led triage.

## Methods

### Study Design and Setting

This study was a retrospective, proof-of-concept evaluation of a RAG-enhanced LLM for ED triage classification. The primary objective was to compare the performance of an RAG-based LLM that referenced both historical case data and the HKAETG against a prompt-only baseline LLM in assigning triage urgency levels.

The HKAETG is a 5-level triage system implemented across all 18 government-funded public EDs in Hong Kong. Its structure is conceptually aligned with other internationally recognized systems such as the MTS and ESI. Under the HKAETG, patients are assigned to 1 of 5 categories based on clinical urgency and time-to-treatment targets: category 1 (critical, 0 minutes), category 2 (emergency, 10 minutes), category 3 (urgent, 30 minutes), category 4 (semiurgent, 120 minutes), and category 5 (nonurgent, 180 minutes).

All study data were sourced from a single center, Princess Margaret Hospital (PMH), a major acute public hospital in Hong Kong and one of the city’s largest tertiary care facilities. PMH operates a 24-hour ED and manages a high volume of complex and time-sensitive cases. In 2023 and 2024, the ED recorded 113,974 and 110,812 visits, respectively, highlighting its central role in emergency care delivery.

### Pilot Study, Sample Size Calculation, and Model Selection

Before the main evaluation, we conducted a pilot study to inform model selection, prompting strategy, and sample size estimation. We randomly sampled 1000 ED triage cases from December 2024; 200 cases were used for pilot testing, and the remaining 800 cases were reserved to simulate a retrieval database. All pilot cases were excluded from the final study.

Three commercially available LLMs (DeepSeek-V3, GPT-4o, and Claude 3.7 Sonnet) were compared in both prompt-only and RAG configurations, with performance assessed against clinician-assigned triage labels using quadratic weighted kappa (QWK) and accuracy. Across models, performance was statistically similar in this pilot. DeepSeek-V3 was therefore selected as the backbone model for the main study primarily on the basis of its inference cost, application programming interface (API) accessibility, and comparable pilot performance.

In the pilot setting, the MECR-RAG configuration achieved an approximate QWK of 0.75 compared with 0.50 for the prompt-only baseline. Using the formula for comparing 2 kappa coefficients described by Fleiss, with α=.05 and power=.80 [20], the minimum required sample size to detect this difference was 48 cases. We nevertheless included 236 consensus-labeled triage cases in the final study to improve the precision of performance estimates and enhance generalizability.

### Data Sources and Participants

The test set comprised ED triage cases collected at PMH between January 1 and December 31, 2023. All ED visits during this period were eligible regardless of age, sex, or presenting problem. To ensure temporal representativeness and reflect real-world variation in case mix, we used a stratified sampling strategy in which 1 calendar day was randomly selected from each month and 20 cases were sampled from that day. Within each sampled day, triage categories were sampled to approximate overall category distributions while modestly oversampling rare categories to improve precision for high-acuity strata. Four cases were excluded because case-number identification errors prevented data retrieval, yielding a final test set of 236 triage cases.

Reference labels were assigned using a blinded, multirater consensus process. Two advanced practice nurses with more than 10 years of emergency experience independently reviewed each case. Raters were blinded to the original nurse-assigned triage category and saw only information available at the time of triage: presenting complaint, condition on arrival, vital signs, and relevant past medical history when documented. Downstream data, including investigations, diagnoses, treatments, and ward progress, were withheld so that labels reflected only the initial triage decision context and matched the information provided to the LLMs. Disagreements were adjudicated by a third senior triage nurse to obtain a final consensus category for each encounter.

For retrieval, we constructed a separate database of 3000 anonymized ED visits to PMH between January 1 and December 31, 2024. All encounters in 2024 were eligible, with no clinical exclusion criteria, to preserve the natural diversity of documentation and presentations. Days were randomly sampled from each month, and cases were then drawn from those days to achieve broad temporal coverage; modest oversampling of rare triage categories (categories 1, 2, and 5) ensured sufficient representation while maintaining the real-world skew toward categories 3 and 4. The final database contained 94/3000 (3.1%) category 1, 120/3000 (4.0%) category 2, 1705/3000 (57.0%) category 3, 1035/3000 (34.5%) category 4, and 46/3000 (1.5%) category 5 encounters, with detailed monthly and category-specific sampling shown in Table S1 in Multimedia Appendix 1. To stress-test temporal robustness and reduce bias from contemporaneous documentation habits, 2023 test cases were evaluated against a 2024 retrieval database using the same triage guideline version; the full rationale is provided in Textbox S1 in Multimedia Appendix 1.

### Preprocessing

Raw, unstructured triage documentation for both test cases and retrieval-database cases was exported from the electronic medical record, deidentified, and converted into a structured, machine-readable text format. We retained only nurse-entered information available at the time of triage (eg, demographics, presenting complaint, condition on arrival, vital signs, and relevant past medical history) and excluded any downstream assessments, investigations, treatments, and outcomes. The latest version of the HKAETG (version 6, revised 2022) was similarly digitized and segmented into retrievable guideline sections. Both triage notes and guideline sections were then processed for semantic retrieval; further preprocessing and summarization details are provided in Textbox S2 in Multimedia Appendix 1.

### LLM and RAG System Description

For all primary analyses, we used DeepSeek‑V3 as the base LLM, accessed via a commercial API without any additional fine-tuning or architectural modification. In what follows, we refer to the DeepSeek‑V3 model without retrieval as the “baseline LLM” and the dual‑source retrieval‑augmented configuration as MECR‑RAG. Within each experimental configuration, the same LLM instance handled all stages of the pipeline (preprocessing, retrieval, and generation) with deterministic generation (temperature=0) to minimize stochastic variability. A separate embedding model was used to generate vector representations for semantic retrieval, and the same embedding configuration was applied during database construction and at query time to ensure consistency.

The pipeline was implemented in Python and executed on a standard desktop workstation without graphics processing unit acceleration. End-to-end processing time for the full MECR‑RAG configuration was approximately 1 minute per case, including preprocessing, retrieval, and triage prediction, suggesting that near-real-time decision support would be technically feasible. Detailed model versions, libraries, and runtime benchmarks are provided in Section A1 in Multimedia Appendix 1.

### MECR‑RAG System Architecture

The MECR‑RAG system follows a 5-stage pipeline designed to mirror how expert triage nurses combine protocol knowledge with experiential reasoning. First, the raw triage note is summarized into a concise, structured representation optimized for retrieval. Second, the model selects the most relevant sections of the HKAETG using an agentic reasoning step that prioritizes life-threatening conditions and documented physiologic abnormalities. Third, the system predicts the most likely attending specialty (eg, medicine, surgery, and orthopedics) to constrain subsequent case retrieval. Fourth, a hybrid retrieval module identifies similar past triage cases by combining metadata filters (age group and predicted specialty) with vector similarity search. Finally, the model applies a structured, MECR prompt that integrates guideline excerpts, retrieved cases, and the index presentation to assign a 5-level urgency category and provide a textual rationale.

**Figure 1:**
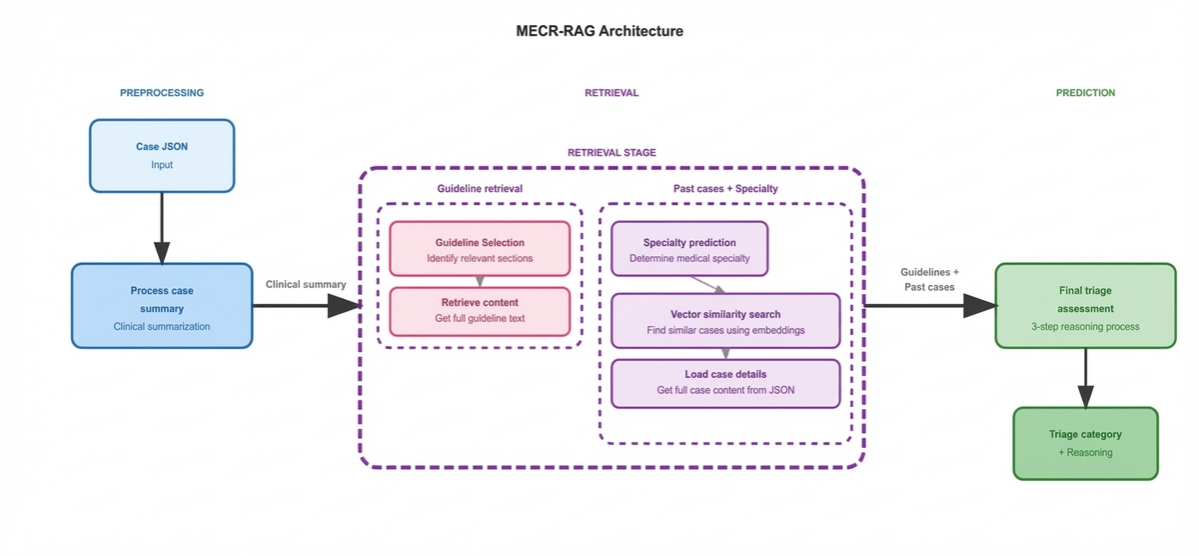
Overview of the Multi‑Evidence Clinical Reasoning retrieval‑augmented generation system architecture. MECR-RAG: Multi‑Evidence Clinical Reasoning retrieval‑augmented generation.

The overall architecture is orchestrated using a modular, node-based workflow that preserves intermediate states, supports full audit trails, and enables systematic component ablation. A schematic overview of the MECR‑RAG pipeline is shown in Figure 1, and additional implementation details are provided in Section A2 in Multimedia Appendix 1.

The MECR‑RAG pipeline consists of 5 sequential processing stages: clinical case summarization, agentic guideline section selection, specialty prediction for metadata filtering, hybrid past-case retrieval combining metadata and vector similarity search, and multievidence reasoning for the final triage assessment. LangGraph nodes manage state transitions and data flow throughout the pipeline.

### Indexing and Database Construction

To support efficient retrieval, both historical triage cases and guideline content were converted into structured, machine-readable representations and indexed in a vector database. Historical cases were transformed into standardized triage-time summaries that captured presenting complaints, key vital signs, and brief clinical context, while preserving links to metadata such as age group, triage category, and attending specialty. The same summarization template was applied to both database cases and incoming test cases to ensure that queries and indexed documents occupied a comparable embedding space.

The HKAETG document was similarly segmented into discrete sections and summarized to highlight the spectrum of presentations covered by each rule set. Each guideline section was stored with a short synopsis and structural metadata (eg, system, chief complaint, and red-flag criteria) to facilitate targeted retrieval during triage. The combined indexing strategy was designed to balance retrieval quality, computational efficiency, and scalability to larger case repositories. Details of prompt templates, sectioning rules, and indexing parameters are described in Section A3 in Multimedia Appendix 1.

### Retrieval System Design

The retrieval layer implements a dual-source strategy that distinguishes authoritative protocol knowledge from experiential case patterns. Guideline retrieval is performed via an agentic selection step: given the summarized index case and a list of guideline section titles and synopses, the LLM selects up to 2 sections it deems most relevant, explicitly favoring conditions associated with high short-term risk or abnormal vital signs. This approach avoids purely lexical matching and aligns guideline selection more closely with clinical reasoning.

Past-case retrieval uses a multistage, hybrid approach. The model first predicts 1 or 2 likely specialties using triage-available information only, then filters the case database by age group and specialty to construct a clinically plausible candidate pool. Within this subset, vector similarity search identifies the closest matches based on the summarized clinical text. A small number of top-ranked cases are returned, along with similarity scores and metadata, for use in the final reasoning step. Similarity thresholds, top-k values, and fallback strategies were chosen empirically and are reported in Section A4 in Multimedia Appendix 1.

### Generation and Prompting Strategy

The generation stage uses an MECR prompt that extends conventional chain-of-thought reasoning for the specific demands of triage. Rather than a single undifferentiated reasoning path, the prompt enforces a 3-tier structure: (1) a clinical risk assessment aligned with formal triage category definitions, (2) integration of condition-specific recommendations from the retrieved HKAETG sections, and (3) comparison with retrieved past cases to identify contextual factors that may support escalation or de-escalation (eg, frailty, social circumstances, and borderline physiology). Each evidence source produces an independent provisional triage recommendation.

The model is then instructed to reconcile these recommendations into a single final category, explicitly justifying the choice and indicating which sources carried the most weight. Outputs include the predicted triage category, a structured explanation, and a coarse confidence label (high, medium, or low), enabling clinicians to audit the reasoning process and gauge when additional scrutiny is warranted. The complete MECR prompt details are provided in Section A5 in Multimedia Appendix 1.

### Ablation Configurations

To isolate the contribution of individual retrieval components, we implemented 3 reduced configurations alongside the full MECR‑RAG system. The baseline configuration used the same triage definition table and reasoning structure but removed all retrieval, relying only on the index case description. The guideline-only configuration added agentic guideline selection but did not use past-case retrieval, whereas the case-only configuration retrieved similar past cases without guideline sections. In all ablation variants, preprocessing steps, LLM model, and evaluation procedures were held constant to ensure that performance differences could be attributed to the presence or absence of specific retrieval sources rather than to implementation artifacts. The details are provided in Section A6 in Multimedia Appendix 1.

### Outcome Definition and Label-Based Evaluation

The primary outcome was the 5-level triage category (categories 1-5) predicted by each LLM configuration, compared with consensus labels assigned by expert triage nurses. Two configurations were evaluated: the MECR‑RAG system, which incorporated both the HKAETG and retrieval of past triage cases, and a prompt-only baseline model with no retrieval component. Model outputs were assessed against the final adjudicated triage category for each case.

The primary performance metric was QWK, chosen because it accounts for the ordinal nature of triage categories and penalizes larger discrepancies more heavily, in line with their greater clinical consequence. Overall accuracy was reported as a complementary measure summarizing the proportion of exact matches between model predictions and consensus labels.

To provide a more clinically grounded view of performance, we also calculated precision, recall, and *F*_1_-score for each triage category and for a post hoc grouping of triage levels into 3 urgency bands: immediate (categories 1 and 2), urgent (category 3), and nonurgent (categories 4 and 5). This mapping reflects local operational workflows, in which small changes in urgency assignment can translate into large differences in waiting time and risk exposure. These group-level metrics were used to characterize overtriage and undertriage patterns beyond aggregate QWK and accuracy estimates.

Because LLM outputs are probabilistic, all primary comparisons between MECR‑RAG and the baseline LLM were repeated across 5 independent runs using identical test cases, prompts, retrieval databases, and API settings. For these analyses, we summarized performance as mean values with 95% CIs calculated across runs. For analyses based on a single representative run—for example, evaluations using the median-performing prediction set or post hoc subgroup comparisons—95% CIs were obtained via nonparametric case-level bootstrapping (1000 resamples). The specific CI method used is reported alongside each result, and full resampling details are provided in Textbox S3 in Multimedia Appendix 1.

### Secondary Outcome-Based Validity Analysis

Because there is no universally accepted gold standard for “true” acuity at ED presentation, triage tools are typically judged against surrogate outcomes such as hospitalization, ICU admission, revisits, and short-term mortality [15,16]. Large observational studies have shown that nurse-assigned categories can substantially underestimate or overestimate severity, with mis-triage affecting up to one-third of patients in some cohorts [17,21]. To complement our consensus label-based evaluation, we therefore derived outcome-based severity tiers and a disposition-safety composite (DSC) and re-examined how MECR-RAG, the baseline LLM, and initial nurse triage aligned with downstream clinical courses.

For all encounters with sufficient follow-up, we assigned an ordinal outcome-based severity tier (R1-R3) adapted from the multilevel reference standard used in MTS validation work, in which a multidisciplinary expert panel defined 3 urgency levels [15]. R1 represented high-acuity courses analogous to the “immediate or very urgent” reference category and was defined by the presence of any marker of life-threatening illness, such as markedly abnormal vital signs with modified early warning score ≥5 or depressed consciousness, emergency surgery or other high-intensity interventions shortly after ED arrival, or death in the ED, ICU, or during the index admission. R2 corresponded to an “urgent” course and included patients who did not meet R1 criteria but required substantial acute treatment (eg, intravenous medication, fluids, and nebulizers) or hospitalization. R3 comprised lower-acuity courses in which none of the R1 or R2 markers were present. Patients who left without being seen (LWBS) were coded separately and excluded from outcome-based binary analyses. Full operational definitions and mapping of specific outcome elements to R1-R3 are provided in Table S2 in Multimedia Appendix 1.

To capture sentinel deterioration events that may reflect underrecognition of severity at triage, we additionally defined a DSC based on components commonly used as ED quality and safety indicators. DSC was coded as positive if any of the following occurred after the index ED visit: (1) unplanned ICU transfer within 72 hours of ward admission, (2) ED revisit within 72 hours resulting in unplanned hospital admission (including ICU admission when present), or (3) all-cause death within 7 days [22-24]. A binary variable, DSC_any, was set to 1 if at least one component was present. Outcome adjudication for R1-R3 and DSC_any was performed using routinely collected outcome data and was fully blinded to the index triage category and all model outputs.

Using these constructs, we prespecified 4 binary analytic “lenses” for sensitivity analyses. A classic lens defined outcome+ as R1 only and outcome– as R2 or R3, with test+ as triage categories 1-2 versus 3-5, mirroring conventional MTS validation [15]. An operational lens defined outcome+ as R1 or R2 versus R3 and test+ as categories 1-3 versus 4-5, reflecting the separation between patients who should not wait in the lowest-priority stream and those who can safely do so. Two augmented lenses incorporated DSC_any: an augmented classic lens (outcome+=R1 or DSC_any; test+=categories 1 and 2) and an augmented operational lens (outcome+=R1 or R2 or DSC_any; test+=categories 1-3). These lenses were intended to test robustness across different, clinically motivated definitions of high severity and to align with prior recommendations on composite triage outcomes. Full definitions are provided in Textbox S4 in Multimedia Appendix 1.

For each lens and each triage method (nurse triage, baseline LLM, and MECR-RAG), we constructed 2×2 tables of test+ versus outcome+ and calculated sensitivity, specificity, and 95% CIs, along with positive and negative likelihood ratios (LR+ and LR–) and diagnostic odds ratios (DORs). To compare models on the same patients, we used paired McNemar tests to assess differences in sensitivity (within outcome+ cases) and specificity (within outcome– cases). As additional clinically interpretable summaries, we examined (1) the proportion of R1 cases assigned to immediate (categories 1 and 2), (2) the proportion of R2 cases assigned to nonurgent (categories 4 and 5), and (3) a weighted harm index per 100 patients that assigns higher penalties to more dangerous errors (R1 or R2 assigned to nonurgent) than to overtriage of R3 cases. Case-level harm scores were compared between models using paired signed tests. Detailed formulas for likelihood ratios, DORs, CIs, and harm index computation are provided in Textbox S5 in Multimedia Appendix 1.

### Statistical Analysis

All analyses were conducted using R (version 4.5.0; R Core Team). Interrater agreement and kappa statistics were calculated using standard packages for reliability analysis. Classification metrics were obtained using established machine learning libraries, and nonparametric CIs were estimated using bootstrap resampling. Between-model comparisons used tests appropriate to the data structure, including Friedman tests with Nemenyi post hoc procedures for repeated-run summary metrics and Cochran Q and McNemar tests for paired binary outcomes. Exploratory subgroup, scaling, and cross-model generalizability analyses were treated as descriptive and were not formally powered for hypothesis testing. Full details of the statistical workflow, including package names, resampling schemes, handling of multiple runs, and exploratory analyses, are provided in Textbox S6 in Multimedia Appendix 1.

### Ethical Considerations

This study was approved by the Hospital Authority Central Institutional Review Board (reference number CIRB-2024-561-4). Because this was a retrospective analysis of anonymized patient records with no impact on clinical care, the Central Institutional Review Board granted a waiver of informed consent. All data used in this study were fully anonymized prior to analysis, and no identifiable patient information was retained or accessible to the study team. No compensation was provided, as no participants were recruited or contacted for this study.

## Results

### Participants

The final test set comprised 236 ED triage encounters from 2023. After expert consensus adjudication, the distribution of triage categories was 23/236 (9.7%) category 1, 23/236 (9.7%) category 2, 82/236 (34.7%) category 3, 98/236 (41.5%) category 4, and 10/236 (4.2%) category 5. The mean patient age was 55.1 (SD 26.2; range 0.3-98.0) years. Overall, 131/236 (55.5%) patients were male and 105/236 (44.5%) were female. The most common clinical specialties were medicine (115/236, 48.7%), orthopedics (36/236, 15.3%), surgery (33/236, 14.0%), and pediatrics (13/236, 5.5%), with the remaining 39/236 (16.5%) classified as other specialties.

The retrieval database used by the MECR‑RAG system consisted of 3000 anonymized ED encounters from 2024. The triage category distribution was constructed to reflect real-world attendance patterns, which are typically skewed toward lower-acuity presentations, with modest upsampling of categories 1, 2, and 5 to ensure adequate representation of rare but clinically important groups. The mean age in the retrieval database was 56.7 (SD 25.6; range 0.2-104.0) years, with an equal sex distribution (1500/3000, 50.0% male and 1500/3000, 50.0% female). The specialty mix was similar to the test set, with medicine accounting for 1395/3000 (46.5%) encounters, followed by orthopedics (441/3000, 14.7%), surgery (435/3000, 14.5%), pediatrics (210/3000, 7.0%), and other specialties (519/3000, 17.3%). Baseline characteristics of both datasets are summarized in Table 1, and characteristics of the 1000‑ and 2000‑case retrieval subsets used in the scaling analysis are provided in Table S3 in Multimedia Appendix 1.

**Table 1:** The demographic and triage category distribution of the test set and 3000-case retrieval-augmented generation database.

Characteristic		Test set (N=236)	3000-case database (N=3000)
Age (years), mean (SD; range)		55.1 (26.2; 0.3-98.0)	56.7 (25.6; 0.2-104.0)
Sex, n (%)			
	Male	131 (55.5)	1500 (50.0)
	Female	105 (44.5)	1500 (50.0)
Triage categories, n (%)			
	Category 1	23 (9.7)	94 (3.1)
	Category 2	23 (9.7)	120 (4.0)
	Category 3	82 (34.7)	1705 (57.0)
	Category 4	98 (41.5)	1035 (34.5)
	Category 5	10 (4.2)	46 (1.5)
Specialty, n (%)			
	Medicine	115 (48.7)	1395 (46.5)
	Orthopedics	36 (15.3)	441 (14.7)
	Surgery	33 (14.0)	435 (14.5)
	Pediatrics	13 (5.5)	210 (7.0)
	Others	39 (16.5)	519 (17.3)

Age is presented as mean (SD). Triage category and specialty values are reported as percentages of total cases within each dataset. Other specialties include dentistry; dermatology; ear, nose, and throat; neurosurgery; obstetrics and gynecology; oncology; ophthalmology; and psychiatry. Both datasets were constructed to reflect real-world triage distributions, with modest oversampling of rare categories (eg, categories 1, 2, and 5) to ensure sufficient representation for model evaluation. Full sampling rationale is provided in the “Methods” section. The test set and retrieval database exhibited broadly comparable distributions in age, sex, and specialty.

### Comparative Performance of MECR‑RAG and the Baseline LLM, Benchmarked Against Expert Rater Agreement

The MECR‑RAG system showed substantially higher agreement with consensus triage labels than the baseline LLM. Across 5 independent runs, MECR‑RAG achieved a mean QWK of 0.902 (SD 0.0021; 95% CI 0.901-0.904), compared with 0.801 (SD 0.004; 95% CI 0.798-0.804) for the baseline model (mean paired difference 0.101, SD 0.0058; 95% CI 0.097-0.106; *P*<.001). Classification accuracy followed the same pattern, with a mean accuracy of 0.802 (SD 0.0082; 95% CI 0.795-0.808) for MECR‑RAG versus 0.542 (SD 0.0073; 95% CI 0.536-0.548) for the baseline LLM (mean paired difference 0.264, SD 0.0150; 95% CI 0.251-0.278; *P*<.001). Similar effect sizes were observed when analyses were repeated using single median‑run prediction sets, indicating that MECR‑RAG’s advantage was robust to run‑to‑run variability.

**Figure 2:**
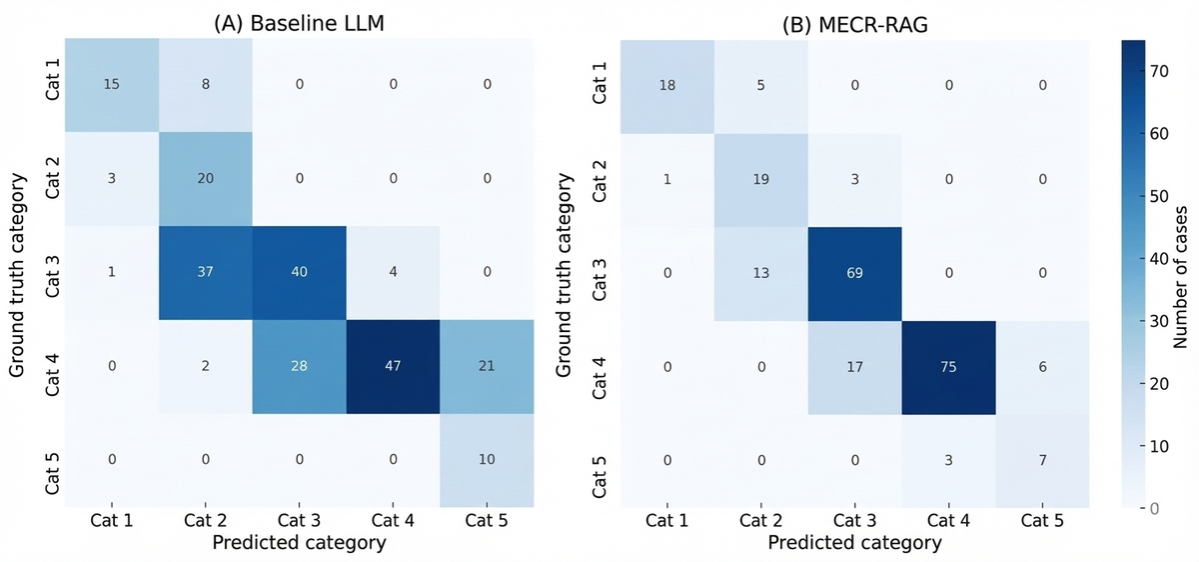
Confusion matrices comparing 5‑level triage classification performance between the baseline prompt‑only large language model (panel A) and the Multi‑Evidence Clinical Reasoning retrieval‑augmented generation system (panel B) on 236 emergency department triage encounters in the test set. LLM: large language model; MERC-RAG: Multi‑Evidence Clinical Reasoning retrieval‑augmented generation.

To benchmark model performance against human experts, we evaluated interrater reliability between the 2 professional triage nurses who generated the reference labels; their QWK was 0.887. By comparison, MECR‑RAG achieved a mean QWK of 0.902 (SD 0.0021; 95% CI 0.901-0.904). Using a prespecified noninferiority margin of −0.05, MECR‑RAG met criteria for noninferiority to expert agreement, and this conclusion was unchanged in sensitivity analyses restricted to single median‑performing runs. Taken together, these findings suggest that MECR‑RAG attains expert‑comparable consistency in 5‑level triage classification while clearly outperforming a prompt‑only LLM. Confusion matrices comparing triage classification performance between the baseline LLM and MECR‑RAG are shown in Figure 2. Results are shown for the median‑performing run across 5 stochastic trials in this figure.

### Performance Using Clinically Grouped Triage Categories

**Figure 3:**
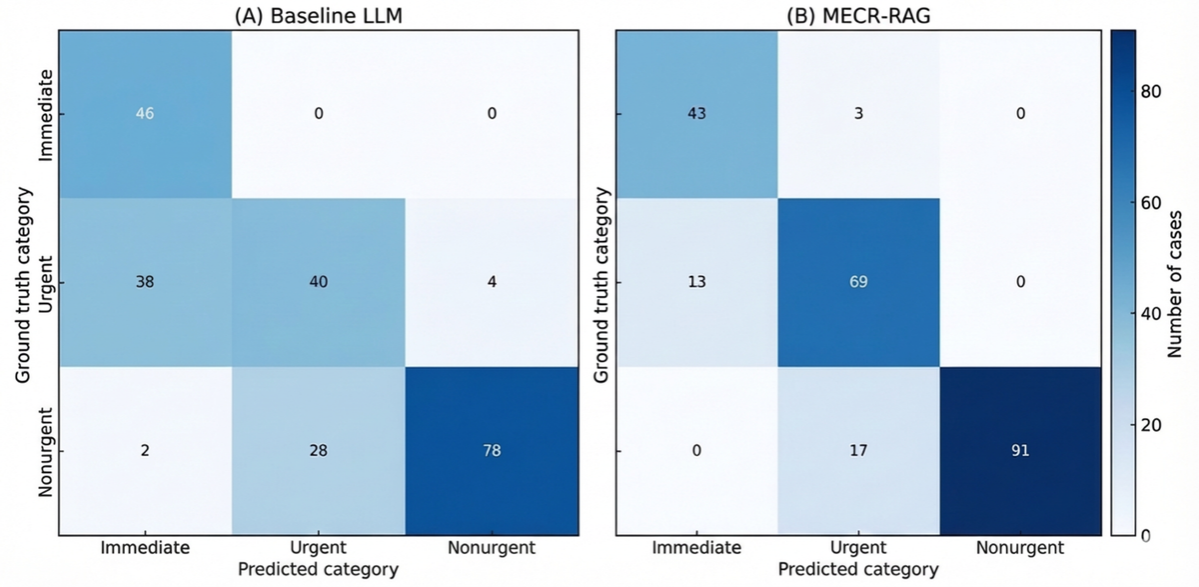
Confusion matrices comparing triage group classification performance between the baseline prompt‑only large language model (panel A) and the Multi‑Evidence Clinical Reasoning retrieval‑augmented generation system (panel B) under a 3‑group schema. LLM: large language model; MERC-RAG: Multi‑Evidence Clinical Reasoning retrieval‑augmented generation.

To reflect operational priorities in the ED, we conducted a post hoc analysis collapsing the 5 triage categories into 3 clinically meaningful groups: immediate (categories 1 and 2), urgent (category 3), and nonurgent (categories 4 and 5). This schema mirrors local workflows, in which immediate cases are seen without delay, urgent cases typically wait approximately 32 minutes, whereas nonurgent cases typically wait approximately 240 minutes based on 2024 institutional data. All metrics in this analysis were derived from the median‑performing model run to approximate single‑run deployment. Confusion matrices for grouped predictions are shown in Figure 3. Results reflect the median‑performing run across 5 stochastic trials in this figure.

Under this 3-group schema, MECR‑RAG maintained high overall performance, achieving a QWK of 0.882 (95% CI 0.840-0.920) compared with 0.759 (95% CI 0.700-0.813) for the baseline LLM (paired difference 0.125, 95% CI 0.077-0.174; *P*<.001). Accuracy similarly favored MECR‑RAG (0.859 vs 0.695; paired difference 0.164, 95% CI 0.110-0.225; *P*<.001). Macrolevel precision, recall, and *F*_1_-score were also higher for MECR‑RAG (precision 0.848 vs 0.691; recall 0.873 vs 0.737; *F*_1_-score 0.860 vs 0.713).

Error-pattern analysis showed that MECR‑RAG slightly reduced undertriage compared with the baseline LLM (3/236, 1.3% vs 4/236, 1.7%; McNemar *P*=.69), while substantially reducing overtriage. Overtriage decreased from 68/236 (28.8%) with the baseline LLM to 30/236 (12.7%) with MECR‑RAG (McNemar *P*<.001). Thus, the model reduced unnecessary escalation without compromising detection of higher-acuity cases.

**Figure 4:**
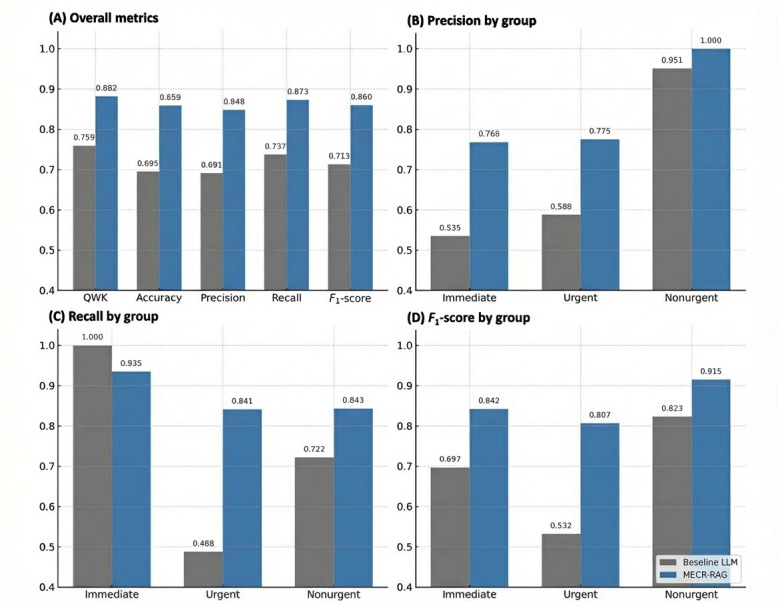
Comparative model performance across overall and triage group-specific metrics. LLM: large language model; MERC-RAG: Multi‑Evidence Clinical Reasoning retrieval‑augmented generation. QWK: quadratic weighted kappa.

Performance advantages for MECR‑RAG were consistent across triage groups. For immediate cases, MECR‑RAG had slightly lower recall than the baseline LLM (0.935 vs 1.000) but substantially higher precision (0.768 vs 0.535), yielding a higher *F*_1_-score (0.842 vs 0.697). Overall correctness did not differ significantly (McNemar *P*=.25), suggesting comparable sensitivity with fewer false positives. In the urgent group, MECR‑RAG markedly outperformed the baseline model (recall 0.841 vs 0.488; precision 0.775 vs 0.588; *F*_1_-score 0.807 vs 0.532; McNemar *P*<.001), indicating improved discrimination in this diagnostically challenging and operationally dominant band. For nonurgent cases, MECR‑RAG again showed higher recall (0.843 vs 0.722) and *F*_1_-score (0.915 vs 0.823), with perfect precision (1.000 vs 0.951; McNemar *P*=.01). These patterns, summarized in Figure 4, suggest that MECR‑RAG delivers a more favorable balance between safety and operational efficiency, particularly in the mid- and low-acuity groups that account for most ED visits and drive waiting-time differentials.

Comparative model performance across overall and triage group-specific metrics. Performance is evaluated using a clinically relevant 3‑level grouping: immediate (categories 1 and 2), urgent (category 3), and nonurgent (categories 4 and 5), corresponding to local time‑to‑physician targets and resource prioritization. Panel A summarizes overall performance across QWK, accuracy, precision, recall, and *F*_1_-score for the baseline prompt‑only LLM and the MECR‑RAG system; MECR‑RAG achieved higher QWK (0.882) and accuracy (0.859) than the baseline LLM (QWK 0.759; accuracy 0.695; both *P*<.001). Panels B-D show precision, recall, and *F*_1_-score, respectively, by triage group, with MECR‑RAG consistently outperforming the baseline LLM across all groups and particularly in the urgent group (*F*_1_-score 0.807 vs 0.532). All results are based on the median‑performing model run.

### Outcome-Informed Validity Relative to Clinical Severity

Among the 236 triage encounters, 226 (95.8%) had sufficient follow‑up information to assign an outcome‑based severity tier (R1**-**R3); the remaining 10 (4.2%) patients were LWBS and were excluded from all outcome‑based analyses but retained in label‑based performance summaries. Within the 226 encounters with outcome labels, 13/226 (5.8%) met R1 criteria, 117/226 (51.8%) met R2 criteria, and 96/226 (42.5%) were classified as R3. When high-severity outcomes were defined as R1+R2 (n=130) and triage “positive” as categories 1**-**3 versus 4**-**5, MECR‑RAG achieved the highest sensitivity for high-risk courses at 124/130 (95.4%), compared with 117/130 (90.0%) for initial nurse triage and 122/130 (93.8%) for the baseline LLM. The gain versus nurses was statistically significant (McNemar *χ*²₁=5.14; *P*=.02), whereas sensitivity did not differ from the baseline LLM (*χ*²₁=0.17; *P*=.68). Specificity for low-risk (R3) cases was 77/96 (80%) for MECR-RAG, 78/96 (81%) for nurses, and 66/96 (69%) for the baseline LLM. Thus, MECR-RAG maintained nurse-level specificity (*χ*²₁=0.00; *P*=1.00) while significantly improving specificity over the baseline LLM (*χ*²₁=5.26; *P*=.02). All 3 systems correctly avoided assigning nonurgent (categories 4 and 5) to any R1 cases (0/13 each). The DOR for distinguishing R1+R2 from R3 was 83.8 (95% CI 32.0**-**218.9) for MECR-RAG, 39.0 (95% CI 18.1**-**84.1) for nurses, and 33.6 (95% CI 14.6**-**77.4) for the baseline LLM, consistent with a more favorable overall trade-off between undertriage and overtriage for MECR-RAG, although CIs overlapped because of limited event counts.

To examine clinically important undertriage, we focused on R2 patients assigned to nonurgent triage (categories 4 and 5). Among 117 R2 encounters, 13/117 (11.1%) were initially labeled nonurgent by nurses, compared with 8/117 (6.8%) by the baseline LLM and 6/117 (5.1%) by MECR-RAG. In paired analysis, MECR-RAG approximately halved R2 nonurgent misclassification relative to nurses (13 to 6 cases; McNemar *χ*²₁=5.14; *P*=.02), with a smaller, nonsignificant reduction compared with the baseline LLM (8 to 6 cases; *χ*²₁=0.17; *P*=.68). Considering the entire triage distribution, MECR-RAG also produced the safest nonurgent queue in terms of hidden high-risk patients: among patients predicted as nonurgent, the proportion with R1+R2 outcomes was 6/83 (7%) for MECR-RAG, compared with 13/91 (14%) for nurses and 8/74 (11%) for the baseline LLM. immediate groups were correspondingly enriched for high-severity outcomes, with 47/47 (100%) R1+R2 for nurses, 56/56 (100%) for MECR-RAG, and 82/86 (95%) for the baseline LLM. These patterns suggest that MECR-RAG reduces undertriage of R2 patients into the lowest-priority stream while preserving a clear outcome-based risk gradient across immediate, urgent, and nonurgent groups.

As a complementary summary of mis-triage burden, we constructed a weighted harm index that assigns higher cost to more dangerous errors: R1 assigned to nonurgent (categories 4 and 5) was weighted as 5, R2 assigned to nonurgent as 2, and R3 assigned to immediate or urgent (categories 1**-**3) as 1. Across the 226 cases with outcome labels, initial nurse triage accumulated a weighted error sum of 44 (harm index 19.47 per 100 patients), the baseline LLM 46 (20.35 per 100 patients), and MECR‑RAG 31 (13.72 per 100 patients). At the individual case level, the baseline LLM incurred higher harm scores than MECR‑RAG in 19 discordant cases and lower scores in 6, with 201 ties (2-sided sign test *P*=.02), indicating a statistically significant net reduction in weighted harm with MECR‑RAG. Compared with nurses, harm scores were lower for MECR‑RAG in 14 cases and higher in 8 (204 ties; *P*=.29), suggesting a numerically favorable but underpowered difference. Overall, these outcome-based analyses indicate that MECR‑RAG not only improves high-severity sensitivity and reduces R2 undertriage but also yields the lowest aggregate mis-triage burden when accounting for both the direction and severity of errors.

To test robustness to alternative definitions of clinical severity, we repeated binary analyses under classic and augmented outcome “lenses” that defined outcome+ using R1 alone, R1 combined with the DSC, and R1 or R2 combined with the composite (see Methods). Across all lenses, MECR‑RAG consistently showed the most favorable trade-off between sensitivity and specificity, with the highest DORs and positive likelihood ratios compared with nurses and the baseline LLM. In a classic Manchester-style lens (outcome+=R1 only; test+=categories 1**-**2 vs 3**-**5), all 3 systems achieved perfect sensitivity for R1 cases, but MECR‑RAG improved specificity relative to the baseline LLM and approached nurse performance. When the DSC (unplanned ICU transfer within 72 hours, 72-hour revisit with admission, or death within 7 days) was incorporated into augmented lenses, MECR‑RAG again maintained the highest discrimination despite wide and overlapping confidence intervals reflecting the small number of sentinel events. Full 2×2 tables, effect estimates, CIs and details for each lens are provided in Tables S4**-**S7 and Section B in Multimedia Appendix 1. Complementary exploratory analyses, including subgroup performance stratified by age and sex and model behavior on adjudicated disagreement cases, are reported in Sections C and D in Multimedia Appendix 1.

### Component-Wise Ablation of Retrieval Mechanisms

To assess the contribution of individual retrieval components, we conducted a component-wise ablation across four configurations: (1) a prompt-only baseline LLM without retrieval, (2) a guideline-only RAG model retrieving sections from the HKAETG, (3) a case-only RAG model retrieving similar past triage cases, and (4) the full MECR‑RAG system combining both guideline and case retrieval. Each configuration used an identical preprocessing and generation pipeline and was evaluated over 5 independent runs. QWK was the primary outcome, with overall accuracy, macrolevel precision, and macrolevel recall as secondary metrics.

**Figure 5:**
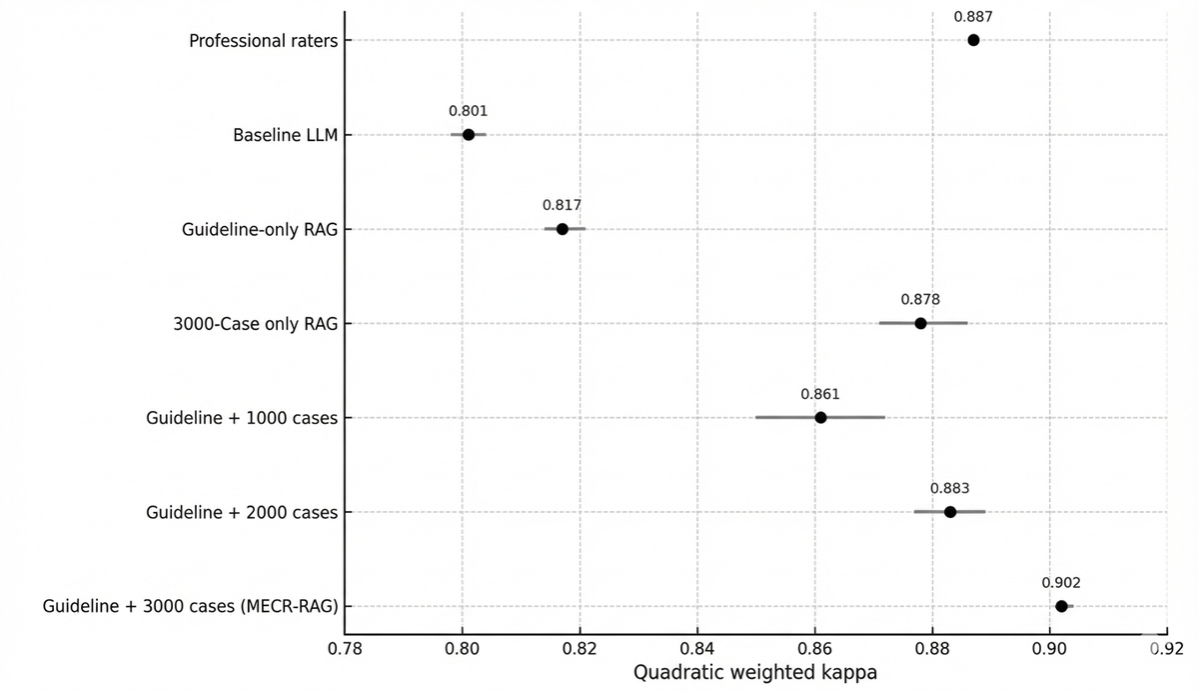
Quadratic weighted kappa performance of model variants and professional raters. LLM: large language model; MERC-RAG: Multi‑Evidence Clinical Reasoning retrieval‑augmented generation; RAG: retrieval‑augmented generation.

**Figure 6:**
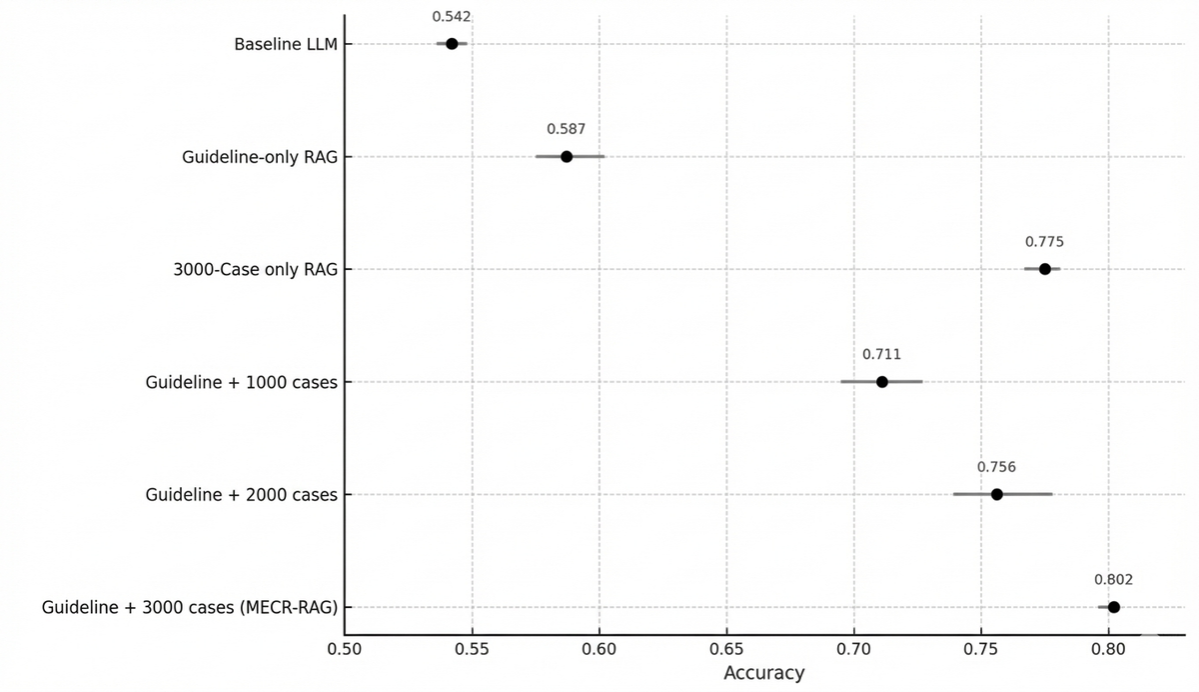
Accuracy performance across model variants. LLM: large language model; MERC-RAG: Multi‑Evidence Clinical Reasoning retrieval‑augmented generation; RAG: retrieval‑augmented generation.

Across configurations, MECR‑RAG achieved the highest agreement with expert consensus (mean QWK 0.902, SD 0.0021; 95% CI 0.901-0.904; accuracy 0.802, SD 0.0082), followed by the case‑only model, then the guideline‑only model, and finally the baseline LLM. Both guideline-based and case-based retrieval improved performance relative to the baseline, but their combined use in MECR‑RAG produced the largest and most consistent gains. Friedman tests on QWK and accuracy across the 4 models were statistically significant, and post hoc Nemenyi comparisons identified MECR‑RAG as the only configuration with a statistically superior rank to the baseline LLM under conservative multiple-comparison control. Full numerical results are shown in Figure 5 (QWK) and Figure 6 (accuracy) and are detailed in Table S8 in Multimedia Appendix 1.

**Figure 7:**
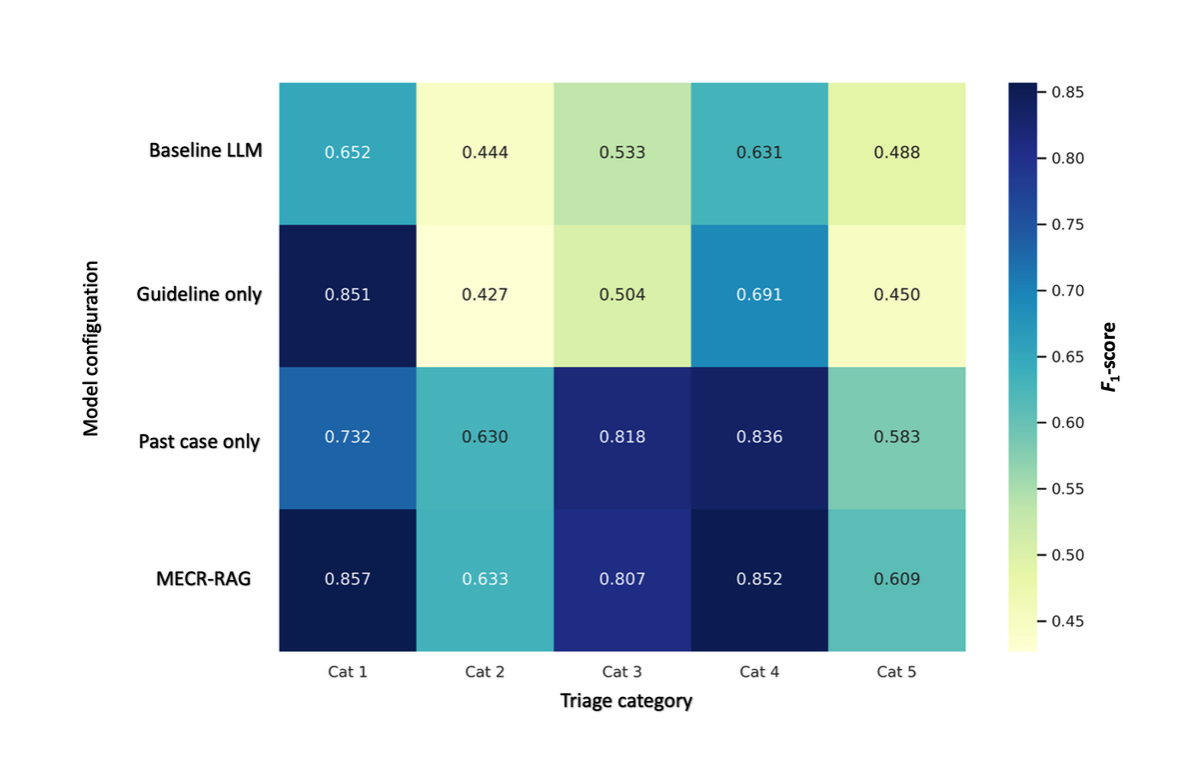
Component-wise ablation analysis of model performance across triage categories. LLM: large language model; MERC-RAG: Multi‑Evidence Clinical Reasoning retrieval‑augmented generation.

To understand how retrieval affected different acuity levels, we examined per-category accuracy and *F*_1_-scores for triage categories 1-5. Guideline retrieval contributed most strongly to category 1 (critical) performance, where the guideline-only model achieved the highest accuracy and *F*_1_-score, consistent with the value of structured, rule-based protocols for recognizing life-threatening presentations. In contrast, case retrieval had the largest impact on categories 3 and 4, where the case-only and MECR‑RAG models substantially outperformed both the baseline and guideline-only configurations, reflecting the importance of experiential pattern recognition in diagnostically ambiguous, midacuity presentations. For categories 2 and 5, model differences were smaller and not statistically robust, although MECR‑RAG generally retained the highest *F*_1_-scores. Overall, MECR‑RAG delivered the best balance of precision and recall across all categories, supporting the complementary value of combining guideline-based and case-based retrieval (Figure 7; detailed estimates in Table S9 in Multimedia Appendix 1).

As an exploratory scaling analysis, we also varied the size of the past-case retrieval database from 1000 to 3000 cases and observed monotonic gains in QWK and accuracy with reduced between-run variability at larger retrieval pools (Tables S3 and S10 in Multimedia Appendix 1).

In Figure 5, the mean QWK values with 95% CIs are shown for the baseline and retrieval‑augmented model variants. Points represent mean QWK across 5 stochastic runs, and horizontal error bars indicate 95% CIs across these runs. The professional benchmark (QWK 0.887) reflects interrater agreement between 2 expert triage nurses. The figure includes both ablation variants—a guideline‑only RAG model and a 3000‑case-only RAG model—and scaling variants that combine guideline and past case retrieval with databases containing 1000, 2000, and 3000 past triage cases. The MECR‑RAG model (guideline plus 3000 past cases) achieved the highest mean QWK and was statistically superior to the baseline prompt‑only large language model (LLM; mean paired difference 0.101, SD 0.0058; 95% CI 0.097-0.106; *P*<.001), while demonstrating noninferior agreement compared with professional raters.

In Figure 6, the mean accuracy values with 95% CIs are shown for the baseline prompt‑only LLM, RAG variants, and combined retrieval configurations. The x‑axis shows accuracy, with points representing mean values across 5 stochastic runs and horizontal error bars indicating 95% CIs. The figure includes ablation variants (guideline‑only and 3000‑case-only RAG) and scaling variants that combine guideline retrieval with 1000, 2000, or 3000 past cases. The MECR‑RAG configuration (guideline plus 3000 past cases) achieved the highest accuracy and demonstrated a statistically significant improvement compared with the baseline LLM.

*F*_1_-scores for each triage category (categories 1-5) across 4 model configurations. Rows represent the baseline prompt‑only LLM, a guideline‑only RAG model, a 3000‑case-only RAG model, and the MECR‑RAG system, and columns represent triage categories. Values reflect outputs from the median‑performing evaluation run. Guideline retrieval contributed most prominently to performance in category 1, while case retrieval improved performance in categories 3 and 4, where patient presentations are more heterogeneous and require contextual interpretation. The MECR‑RAG system generally achieved the highest or near‑highest *F*_1_-scores across triage categories, illustrating the additive value of combining structured guideline knowledge with experiential case‑based retrieval.

### Generalizability Across LLM Architectures

The primary model used for all prior evaluations was DeepSeek‑V3. To evaluate whether the MECR‑RAG framework generalizes beyond this development model, we conducted exploratory assessments using 2 additional foundation models: Claude 3.7 and GPT‑4o. Each model was tested on the same 236‑case triage dataset with the same 3000‑case retrieval database under 2 configurations: the MECR‑RAG system and a baseline, prompt‑only LLM without retrieval augmentation.

In these single‑run evaluations, MECR‑RAG consistently outperformed the corresponding baseline configuration. For Claude 3.7, QWK improved from 0.840 under the baseline to 0.890 with MECR‑RAG. Similarly, GPT‑4o achieved a QWK of 0.890 with MECR‑RAG, compared with 0.778 without retrieval. Although these results are based on single inference runs and are not statistically powered for formal comparison, they provide preliminary evidence that the dual‑source retrieval framework may confer similar relative gains across distinct LLM back ends. A detailed breakdown of model predictions versus consensus triage labels for Claude 3.7 and GPT‑4o, under both MECR‑RAG and baseline conditions, is provided in Figures S1 and S2 in Multimedia Appendix 1, respectively.

Taken together, these preliminary findings suggest that the benefits of dual‑source retrieval generalize beyond DeepSeek‑V3 and may confer cross‑model robustness of MECR‑RAG across LLMs from different developers, supporting its broader applicability in diverse clinical settings. Further benchmarking across a wider range of model families, input formats, and deployment environments is warranted to validate robustness and assess trade‑offs in latency, interpretability, and resource efficiency for real‑world clinical deployment.

## Discussion

### Principal Findings

This retrospective evaluation suggests that a dual-source, retrieval-augmented LLM can achieve expert-comparable agreement with consensus nurse triage labels while substantially outperforming a prompt-only baseline model. MECR‑RAG improved QWK and accuracy over the baseline LLM under both the 5‑level and 3‑group schemas and met prespecified noninferiority criteria compared with professional triage nurses. The largest gains occurred in Categories 3 and 4 and in the urgent versus nonurgent grouping, which together accounted for most ED visits and are operationally dominant, with large differences in waiting times. Ablation analyses indicated that both guideline retrieval and case retrieval contributed to performance, with the full dual‑source MECR‑RAG configuration consistently outperforming guideline‑only and case‑only variants. Performance also improved as the retrieval database increased from 1000 to 3000 cases, suggesting that retrieval quality benefits from a richer case pool. Exploratory cross‑model experiments with Claude 3.7 Sonnet and GPT‑4o further suggested that these benefits generalize across different LLM back ends.

### Ground Truth Validity and Construct Interpretation

In this study, the primary reference standard was expert nurse consensus at the time of presentation, which reflects routine clinical decision-making but is an imperfect proxy for underlying acuity. Large US data on ESI version 4 report mis-triage in 32.2% of encounters (3.3% undertriage and 28.9% overtriage), underscoring that nurse-level labels are imperfect proxies for “true” acuity [17]. Even with trained emergency staff, approximately 17% of triage assignments may be inaccurate [21]. To address these limitations, we complemented consensus labels with outcome-informed constructs, ordinal severity tiers (R1-R3) and a DSC, adapted from MTS validation work and recommendations on triage outcome selection [15,22,24].

Within these outcome-based frameworks, MECR‑RAG showed higher sensitivity for R1+R2 courses than initial nurse triage at comparable specificity, reduced misclassification of R2 patients into the nonurgent stream, and yielded the lowest weighted harm index, suggesting closer alignment between assigned urgency and downstream clinical course. At the same time, event counts for the most severe outcomes were modest, and both nurse consensus and the composite outcomes remain pragmatic proxies rather than definitive measures of “true” severity. We therefore interpret these findings as supportive evidence that MECR‑RAG performs well against clinically grounded, outcome‑informed constructs of acuity, rather than as proof that it fully captures an underlying severity state.

### Comparison With Prior Work

To our knowledge, this is the first study to evaluate a dual‑source, RAG‑enhanced LLM for emergency triage using raw triage documentation paired with expert‑consensus reference labels and complementary outcome‑informed severity constructs. Prior RAG studies in emergency care have shown that retrieval can improve triage or referral performance on simulated exam‑style cases or curated ED datasets but have relied on structured scenarios or routine triage labels without independent adjudication [13,14]. Our design addresses the broader evidence gap noted in a recent systematic review, in which only 5% of 519 published evaluations of health care LLMs used real patient care data for testing [25]. This provides a more realistic and stringent test of RAG‑enhanced triage than has been reported previously.

Masanneck et al [4] established an early benchmark by evaluating LLM triage performance against expert assessments, but their dataset consisted of physician‑curated vignettes from a single day, with selected patient attributes adjusted according to predefined standard operating procedures. In contrast, our study applies dual‑source RAG to a year‑long sample of anonymized triage notes without manual editing or reinterpretation, preserving the fidelity of frontline documentation and temporal variation. Zaboli et al [3] and Paslı et al [7] have likewise emphasized the importance of handling unstructured narratives and integrating local triage frameworks; our dual‑retrieval design directly addresses these needs by combining structured guideline content with real‑world case patterns.

Our findings also align with broader work on nurse triage systems, showing that midacuity presentations are both the most common and the most difficult to classify. In a large cohort of over 5.3 million adult ED encounters using ESI version 4, Sax et al [17] found that approximately 29% of visits were overtriaged and that the sensitivity of ESI version 4 for identifying low‑acuity, low‑resource patients (correctly assigning ESI 4-5 among patients using <2 resources and with no critical interventions) was only 50%. A majority of encounters were assigned to the midlevel category ESI 3, highlighting poor differentiation between lower‑ and higher‑acuity patients. Validation of the HKAETG has similarly shown the lowest interrater agreement for categories 3 and 4, reflecting the intrinsic difficulty of distinguishing “urgent” from “semiurgent” presentations [26]. In our cohort, categories 3 and 4 comprised about three‑quarters of all cases, and the prompt‑only LLM reproduced the overtriage patterns documented in both human and LLM triage studies. In contrast, MECR‑RAG substantially reduced overtriage and improved precision and recall specifically in these mid‑ and low‑acuity strata, suggesting that dual‑source retrieval may help address a long‑standing bottleneck in emergency triage performance.

### Clinical and Operational Implications

Although we did not directly measure ED length of stay (LOS), boarding, or mortality, several findings suggest how MECR‑RAG could plausibly influence safety and flow if implemented as decision support. Under a 3‑group schema (immediate, urgent, and nonurgent), MECR‑RAG achieved higher agreement and accuracy than the prompt‑only LLM, with markedly less overtriage and similarly low undertriage. Gains were concentrated in the urgent and nonurgent tiers, which dominate the case mix and are associated with substantially longer waits for lower‑priority patients at our center. In outcome‑based analyses, MECR‑RAG showed higher sensitivity for R1+R2 courses than initial nurse triage at nurse‑level specificity, reduced misclassification of R2 patients into the nonurgent stream, and yielded the lowest severity‑weighted harm index. Together, these patterns indicate fewer high‑risk patients “hidden” in the longest‑wait queue and fewer low‑risk patients unnecessarily occupying high‑acuity resources, aligning urgency assignments more closely with downstream clinical course.

These redistributions of risk are clinically relevant because prolonged ED LOS, crowding, and boarding have been repeatedly linked to delayed treatment, longer inpatient stays, and higher short‑term mortality. More efficient separation between urgent and nonurgent queues could, in principle, help decompress monitored areas, reduce avoidable time in high‑acuity spaces for low‑risk patients, and support more timely care for those with high‑severity courses. However, our evaluation was retrospective and did not include LOS, crowding, or mortality as prespecified end points. Any potential contribution of MECR‑RAG therefore remains hypothesis‑generating and must be tested in prospective, outcome‑focused studies.

**Figure 8:**
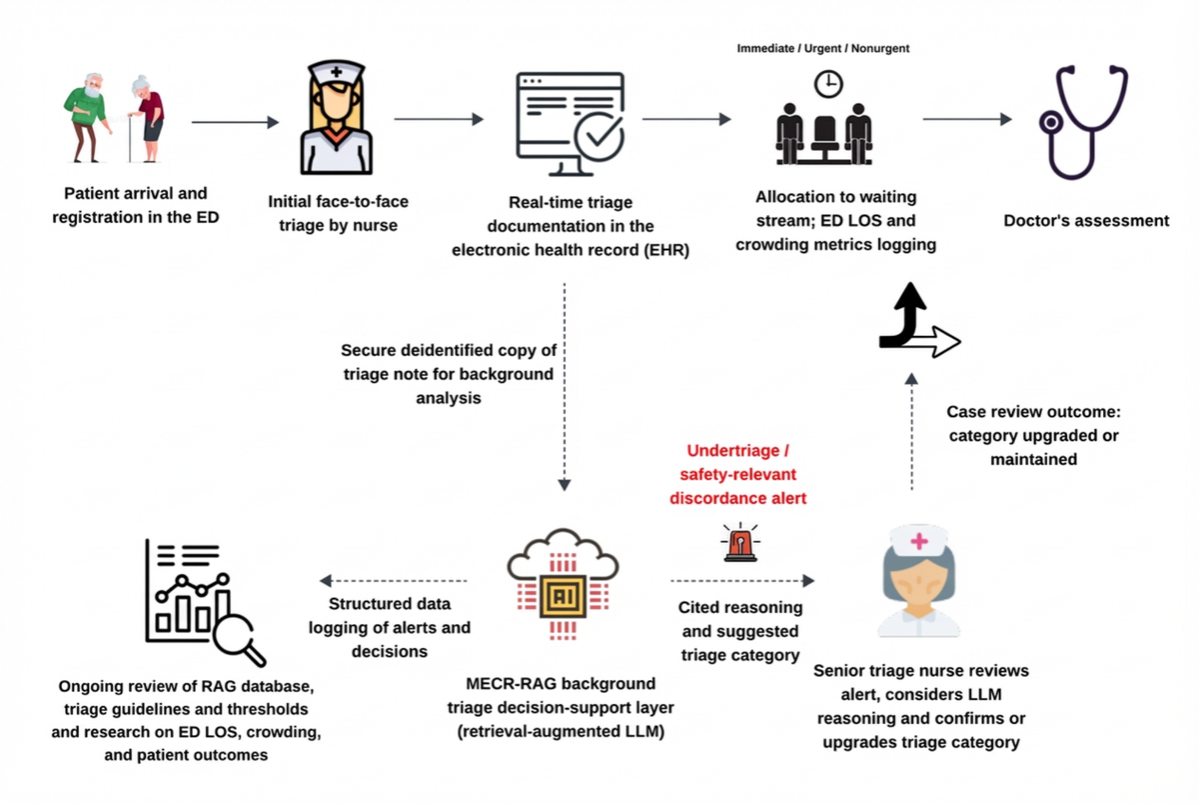
Conceptual human‑in‑the‑loop workflow for Multi-Evidence Clinical Reasoning retrieval-augmented generation as a background triage decision‑support layer in the emergency department. ED: emergency department; LLM: large language model; LOS: length of stay; MECR-RAG: Multi-Evidence Clinical Reasoning retrieval-augmented generation; RAG: retrieval-augmented generation.

As illustrated in the proposed human‑in‑the‑loop workflow (Figure 8), MECR‑RAG is intended to function as a background triage decision‑support layer: triage nurses retain primary responsibility for assigning categories, while a secure, deidentified copy of the triage note is analyzed in parallel, with the system remaining silent for most encounters and surfacing alerts only for predefined safety‑relevant discordances (eg, potential undertriage of urgent or immediate cases). Alerts and explanations can then be reviewed by a senior nurse, who may confirm or upgrade the triage category, while all alerts, decisions, and associated operational metrics are logged to support ongoing quality assurance and research on ED LOS, crowding, and downstream outcomes. A staged implementation strategy, beginning with shadow‑mode deployment and progressing to focused pilots of discordance alerts with monitoring for automation bias, alert fatigue, throughput, and equity, will be essential before wider rollout.

Patients follow the routine care pathway shown in the top row: arrival and registration in the ED, initial face‑to‑face triage by a nurse, real‑time triage documentation in the electronic health record, allocation to an urgency‑based waiting stream (immediate, urgent, or nonurgent) with logging of ED LOS and crowding metrics, and subsequent doctor’s assessment. In parallel, a secure deidentified copy of the triage note is sent to the MECR‑RAG system, which operates as a background triage decision‑support layer. MECR‑RAG generates its own triage recommendation with cited reasoning and remains silent for most encounters, but triggers an undertriage or other safety‑relevant discordance alert when its recommendation conflicts with the nurse‑assigned category. Alerts and explanations are routed to a senior triage nurse, who may confirm or upgrade the triage category; all alerts, decisions, and outcomes are logged to support ongoing quality assurance and research on ED LOS, crowding, and patient‑centered outcomes.

### Limitations and Future Directions

This study has several important limitations. First, within the staged evaluation pathway outlined by DECIDE‑AI (Developmental and Exploratory Clinical Investigations of Decision support systems driven by Artificial Intelligence), this retrospective, offline prediction study represents preclinical (in silico) validation and should not be interpreted as evidence of real‑world clinical effectiveness or safety [27]. Moreover, strong offline discrimination does not reliably translate into clinical benefit after implementation; prior early warning and sepsis tools (including the Rothman Index and the Epic Sepsis Model) have shown materially weaker performance and limited impact in independent, postdeployment evaluations, partly due to differences in data quality, missingness, case mix, alert logic, and workflow across sites [28-32]. Retrospective deep learning models are also vulnerable to dataset shift, class imbalance, and missing data, further limiting transportability across settings and time [33]. Randomized evaluations of AI‑enabled interventions similarly suggest that even high‑performing models may fail to improve primary clinical end points without careful workflow integration and attention to system constraints [34,35]. Accordingly, our retrospective, single‑center results for MECR‑RAG should be viewed as predeployment evidence and require prospective, multicenter evaluation before inferring equivalent real‑time performance or improved patient outcomes.

Second**,** the evaluation was conducted in a single ED using the HKAETG; performance may differ under other triage frameworks (eg, MTS and ESI), languages, documentation practices, workflows, and patient case mix. Although the retrieval‑augmented design is modular and could, in principle, be adapted by reindexing local guidelines and locally representative triage notes without retraining the underlying LLM, portability remains an empirical question and requires external validation and prospective multicenter evaluation.

Third, the retrieval database and text summaries were constructed automatically from routine triage notes without manual adjudication, and prompts were generated without clinician editing. This reflects realistic deployment conditions and improves scalability, but some retrieved exemplars may be mislabeled or incomplete, and clinically relevant nuances may be lost during summarization. In addition, we did not directly evaluate usability, interpretability, automation bias, or workflow impact, which are increasingly recognized as critical determinants of the safety and effectiveness of AI‑enabled clinical decision-support systems [32,36].

These limitations point to clear priorities for future work. Prospective, multicenter studies across institutions using different triage frameworks (HKAETG, MTS, and ESI) are needed to assess generalizability and to determine how MECR‑RAG performs in real‑time workflows. Consistent with DECIDE‑AI and recent guidance from major journals and professional societies, future evaluations should move beyond agreement metrics to prespecify patient‑centered and operational end points, including ED and hospital LOS, crowding and boarding times, unexpected ICU transfer, time‑critical treatment delays, and short‑term mortality [18,37-39]. Implementation‑focused studies should also examine how MECR‑RAG is integrated into practice (eg, as a second reader or discordance alert), its effects on clinician behavior, workload, and equity, and unintended consequences such as alert fatigue or overreliance on model outputs, ideally using pragmatic randomized or quasi‑experimental designs where feasible [34,35]. Framing this work as an early‑phase evaluation and explicitly outlining the need for prospective, outcome‑focused trials in line with current AI evaluation guidance positions MECR‑RAG as a promising candidate for further study rather than a tool ready for immediate widespread deployment.

### Conclusions

In summary, this retrospective, single‑center study provides one of the first evaluations of a dual‑source, retrieval‑augmented LLM for emergency triage on real triage notes with expert consensus labels, extending prior work beyond simulated scenarios and routine labels. MECR‑RAG achieved agreement comparable to expert nurses and clearly outperformed a prompt‑only LLM, particularly in the high‑volume category 3-4 bands, while approximately halving overtriage and keeping undertriage very low. In exploratory outcome‑based analyses within this cohort, MECR‑RAG also showed higher sensitivity for high‑severity courses, a safer nonurgent queue, and the lowest severity‑weighted harm index compared with initial nurse triage. These findings support the potential of retrieval‑augmented LLMs as clinically coherent decision‑support tools that function as background second readers—surfacing guidelines and analogous cases, flagging discordant or high‑risk assignments for senior review—and thereby potentially improving allocation of high‑acuity resources in ways that could, if replicated prospectively, help reduce ED crowding, LOS, and related adverse outcomes. However, this remains an early‑phase, retrospective, single‑center evaluation based on proxy outcomes, and rigorous prospective multicenter studies across different triage frameworks, with prespecified patient‑centered and operational end points and careful attention to workflow, usability, and equity, are essential before such systems can be recommended for routine clinical deployment.

## Appendix

Multimedia Appendix 1 Detailed Multi-Evidence Clinical Reasoning retrieval‑augmented generation implementation, prompts, and additional analyses.

## Abbreviations

AI: artificial intelligence
API: application programming interface
DECIDE-AI: Developmental and Exploratory Clinical Investigations of Decision support systems driven by Artificial Intelligence
DOR: diagnostic odds ratio
DSC: disposition-safety composite
ED: emergency department
ESI: Emergency Severity Index
HKAETG: Hong Kong Accident and Emergency Triage Guidelines
ICU: intensive care unit
JAMA: Journal of the American Medical Association
LLM: large language model
LOS: length of stay
LWBS: left without being seen
MECR: Multi-Evidence Clinical Reasoning
MECR-RAG: Multi-Evidence Clinical Reasoning retrieval-augmented generation
MIMIC‑IV: Medical Information Mart for Intensive Care IV
MTS: Manchester Triage System
PMH: Princess Margaret Hospital
QWK: quadratic weighted kappa
RAG: retrieval-augmented generation
TRIPOD-LLM: Transparent Reporting of a multivariable prediction model for Individual Prognosis Or Diagnosis - Large Language Models

## Data Availability

The datasets generated or analyzed during this study are not publicly available due to institutional and regulatory restrictions imposed by the Hospital Authority of Hong Kong, but are available from the corresponding author on reasonable request. The underlying code supporting the findings of this study is available at MECR-RAG-Clinical-Research [40].
