# Supplementary material for: Multi-Evidence Clinical Reasoning With Retrieval-Augmented Generation for Emergency Triage: Retrospective Evaluation Study
Source: JMIR Med Inform. 2026 Jan 26;14:e82026. doi: 10.2196/82026 (PMC12887567; doi:10.2196/82026)
Supplement: Multimedia Appendix 1 [file medinform_v14i1e82026_app1.docx]

**Multimedia Appendix 1. Detailed MECR‑RAG implementation, prompts, and supplementary analyses**

**Tables (in Order of Appearance)**

**Table S1. Distribution of category in the 3000 database sampled across 12 months in 2024.**

Monthly breakdown of the 3,000-case RAG retrieval database used in this study. Seven random days were sampled per calendar month from 1 January to 31 December 2024, with 240–260 anonymised patient attendances drawn per month. Triage categories were not fixed during sampling but followed natural month-to-month variation in clinical casemix.

| Month | Category 1 | Category 2 | Category 3 | Category 4 | Category 5 | Total |
| --- | --- | --- | --- | --- | --- | --- |
| Jan | 5 | 12 | 141 | 85 | 4 | 247 |
| Feb | 7 | 10 | 142 | 83 | 4 | 246 |
| Mar | 5 | 9 | 141 | 83 | 4 | 242 |
| Apr | 5 | 9 | 141 | 85 | 3 | 243 |
| May | 6 | 10 | 142 | 90 | 5 | 253 |
| Jun | 6 | 11 | 140 | 85 | 3 | 245 |
| Jul | 9 | 9 | 146 | 85 | 3 | 252 |
| Aug | 13 | 9 | 144 | 87 | 4 | 257 |
| Sep | 11 | 13 | 141 | 87 | 4 | 256 |
| Oct | 10 | 10 | 143 | 88 | 4 | 255 |
| Nov | 7 | 10 | 145 | 89 | 4 | 255 |
| Dec | 10 | 8 | 139 | 88 | 4 | 249 |
| Total | 96 | 120 | 1704 | 1035 | 45 | 3000 |

**Table S2. Operational mapping of outcomes to severity tiers R1–R3.** These definitions were informed by Manchester Triage System validation studies and recent methodological work on selecting appropriate triage outcomes

| **Tier** | **Corresponding MTS urgency** | **Approximate maximum waiting time** | **Adult outcome markers (any of)** | **Paediatric outcome markers (any of)** |
| --- | --- | --- | --- | --- |
| R1 | Immediate and Very urgent | 0–10 minutes | • Markedly abnormal vital signs defined by high modified early-warning score >=5  • Depressed level of consciousness (reacts only to pain or unresponsive)  • Death in the ED, ICU, or high-dependency/step-up unit admission  • Emergency surgery within 4 hours of arrival (including urgent cardiac or major vascular procedures) | • Markedly abnormal paediatric vital signs according to a previously used reference standard based on the pediatric risk of mortality score (PRISM III)  • Depressed level of consciousness (reacts only to pain or unresponsive)  • Death in the ED or ICU admission |
| R2 | Urgent | ~60 minutes | • Intravenous medication, fluids, or nebulisers administered in the ED  • Unplanned hospital admission from the ED (ward level) | • Intravenous medication, fluids, or nebulisers administered in the ED  • Unplanned hospital admission from the ED |
| R3 | Standard and Non-urgent | 120–240 minutes | • None of the above | • None of the above |

**Excluded category**

Left without being seen (LWBS): patients who left the ED before medical assessment or treatment. LWBS cases were analysed descriptively but excluded from outcome-based binary comparisons.

**Table S3. Demographic and triage category distribution of the 1,000- and 2,000-case retrieval databases.**

Summary statistics of the 1,000- and 2,000-case retrieval databases used in the scaling analysis. Age is reported as mean (SD). Triage categories and specialties are expressed as percentages of the total case volume for each dataset.

| Characteristic | 1,000-case Database | 2,000-case Database |
| --- | --- | --- |
| Age, mean (SD) | 57.2 (25.4) | 56.4 (25.6) |
| Sex – Male (%) | 48.3 | 49.9 |
| Sex – Female (%) | 51.7 | 50.1 |
| Triage Category 1 (%) | 3.2 | 3.2 |
| Triage Category 2 (%) | 4.0 | 4.0 |
| Triage Category 3 (%) | 57.1 | 57.1 |
| Triage Category 4 (%) | 34.2 | 34.2 |
| Triage Category 5 (%) | 1.5 | 1.5 |
| Specialty – Medicine (%) | 45.6 | 45.8 |
| Specialty – Orthopaedics (%) | 14.7 | 14.8 |
| Specialty – Surgery (%) | 14.6 | 14.6 |
| Specialty – Paediatrics (%) | 6.8 | 7.2 |
| Specialty – Others (%) | 18.3 | 17.6 |

**Table S4.** Operational binary performance (Test+ = Categories 1–3; Outcome+ = R1+R2; Outcome– = R3; known outcomes only)

| Model | TP | FN | TN | FP | Sensitivity, % (95% CI) | Specificity, % (95% CI) | LR+ | LR− | DOR |
| --- | --- | --- | --- | --- | --- | --- | --- | --- | --- |
| Nurse | 117 | 13 | 78 | 18 | 90.0 (83.5–94.6) | 81.2 (72.0–88.5) | 4.80 | 0.123 | 39.0 |
| Baseline LLM | 122 | 8 | 66 | 30 | 93.8 (88.2–97.3) | 68.8 (58.5–77.8) | 3.00 | 0.09 | 33.5 |
| MECR-RAG | 124 | 6 | 77 | 19 | 95.4 (90.2–98.3) | 80.2 (70.8–87.6) | 4.82 | 0.058 | 83.8 |

Operational binary performance for nurse triage, baseline LLM, and MECR‑RAG.
Test+ is defined as triage Categories 1–3 versus 4–5 (Non‑Urgent), and Outcome+ as composite high‑severity R1+R2 versus R3 (known outcomes only). The table reports 2×2 counts (TP, FN, TN, FP), sensitivity and specificity with 95% CIs, and derived likelihood ratios and diagnostic odds ratios.

**Table S5.** Classic Manchester Triage–style binary performance (Test+ = Categories 1–2; Outcome+ = R1; Outcome– = R2+R3; known outcomes only)

| Model | TP | FN | TN | FP | Sensitivity, % (95% CI) | Specificity, % (95% CI) | LR+ | LR− | DOR |
| --- | --- | --- | --- | --- | --- | --- | --- | --- | --- |
| Nurse | 13 | 0 | 179 | 34 | 100.0 (75.3–100.0) | 84.0 (78.4–88.7) | 6.26 | 0 | — |
| Baseline LLM | 13 | 0 | 140 | 73 | 100.0 (75.3–100.0) | 65.7 (58.9–72.1) | 2.92 | 0 | — |
| MECR-RAG | 13 | 0 | 170 | 43 | 100.0 (75.3–100.0) | 79.8 (73.8–85.0) | 4.95 | 0 | — |

Classic Manchester Triage–style binary performance.
Test+ is defined as triage Categories 1–2 versus 3–5, and Outcome+ as R1 (most severe) versus R2+R3 (lower severity). All models achieve perfect sensitivity for R1; the table highlights differences in specificity and likelihood ratios across nurse triage, the baseline LLM, and MECR‑RAG.

**Table S6.** Augmented classic binary performance (Test+ = Categories 1–2; Outcome+ = R1 or DSC; known outcomes only)

| Model | TP | FN | TN | FP | Sensitivity, % (95% CI) | Specificity, % (95% CI) | LR+ | LR− | DOR |
| --- | --- | --- | --- | --- | --- | --- | --- | --- | --- |
| Nurse | 21 | 6 | 173 | 26 | 77.8 (57.7–91.4) | 86.9 (81.4–91.3) | 5.95 | 0.256 | 23.3 |
| Baseline LLM | 26 | 1 | 139 | 60 | 96.3 (81.0–99.9) | 69.8 (63.0–76.1) | 3.19 | 0.053 | 60.2 |
| MECR-RAG | 26 | 1 | 169 | 30 | 96.3 (81.0–99.9) | 84.9 (79.2–89.6) | 6.39 | 0.044 | 146.5 |

Augmented classic binary performance incorporating the disposition‑safety composite (DSC).
Test+ is defined as Categories 1–2 versus 3–5, and Outcome+ as R1 or any DSC event (unplanned ICU transfer, early readmission, or short‑term mortality). The table summarises how each system balances sensitivity for this expanded high‑risk group against specificity for remaining encounters.

**Table S7.** Augmented operational binary performance (Test+ = Categories 1–3; Outcome+ = R1 or R2 or DSC; known outcomes only)

| Model | TP | FN | TN | FP | Sensitivity, % (95% CI) | Specificity, % (95% CI) | LR+ | LR− | DOR |
| --- | --- | --- | --- | --- | --- | --- | --- | --- | --- |
| Nurse | 118 | 13 | 78 | 17 | 90.1 (83.6–94.6) | 82.1 (72.9–89.2) | 5.03 | 0.121 | 41.6 |
| Baseline LLM | 122 | 9 | 65 | 30 | 93.1 (87.4–96.8) | 68.4 (58.1–77.6) | 2.95 | 0.1 | 29.4 |
| MECR-RAG | 125 | 6 | 77 | 18 | 95.4 (90.3–98.3) | 81.1 (71.7–88.4) | 5.04 | 0.057 | 89.1 |

Augmented operational binary performance incorporating the disposition‑safety composite (DSC).
Test+ is defined as Categories 1–3 versus 4–5, and Outcome+ as R1 or R2 or any DSC event. This table shows triage performance when both intermediate‑severity outcomes and sentinel deterioration events are treated as high‑risk, providing a robustness check of MECR‑RAG’s advantage over nurse triage and the baseline LLM.

**Table S8. Overall quadratic weighted kappa (QWK), accuracy, macro-precision, and macro-recall for ablation configurations (five runs).**

| Model | QWK (mean) | QWK, 95% CI | Accuracy | Macro-precision | Macro-recall | ΔQWK vs baseline, mean | ΔQWK, 95% CI | *P* value |
| --- | --- | --- | --- | --- | --- | --- | --- | --- |
| Baseline LLM (prompt-only) | 0.801 | 0.7976–0.8037 | 0.542 | 0.584 | 0.698 | 0.000 | Reference | — |
| Guideline-only RAG | 0.817 | 0.8137–0.8210 | 0.585 | 0.589 | 0.716 | 0.016 | 0.0146–0.0174 | *P*<.001 |
| Case-only RAG | 0.878 | 0.8714–0.8857 | 0.784 | 0.717 | 0.745 | 0.077 | 0.0724–0.0820 | *P*<.001 |
| MECR-RAG (full dual-source) | 0.902 | 0.9007–0.9038 | 0.797 | 0.747 | 0.783 | 0.101 | 0.0971–0.1062 | *P*<.001 |

^a^ QWK differences and 95% confidence intervals (CIs) for Guideline-only and Case-only versus the baseline LLM are from the ablation bootstrap analysis; MECR-RAG versus baseline uses the paired bootstrap estimate from the 3,000-case comparison**Table S9.** Per‑category accuracy and F1 scores for triage Categories 1–5 across ablation configurations

**Table S9. Per-category accuracy and F1 scores for triage Categories 1–5 across ablation configurations (median-performing run).**

| Category | Model | Accuracy | F1 score |
| --- | --- | --- | --- |
| 1 | Baseline LLM | 0.6522 | 0.6522 |
| 1 | MECR-RAG | 0.7826 | 0.8572 |
| 1 | Guideline-only | 0.8696 | 0.8511 |
| 1 | Case-only | 0.6522 | 0.7317 |
| 2 | Baseline LLM | 0.8696 | 0.4444 |
| 2 | MECR-RAG | 0.8261 | 0.6333 |
| 2 | Guideline-only | 0.8261 | 0.4270 |
| 2 | Case-only | 0.7391 | 0.6296 |
| 3 | Baseline LLM | 0.4878 | 0.5333 |
| 3 | MECR-RAG | 0.8415 | 0.8070 |
| 3 | Guideline-only | 0.4024 | 0.5038 |
| 3 | Case-only | 0.8780 | 0.8180 |
| 4 | Baseline LLM | 0.4796 | 0.6309 |
| 4 | MECR-RAG | 0.7653 | 0.8523 |
| 4 | Guideline-only | 0.5816 | 0.6909 |
| 4 | Case-only | 0.7551 | 0.8362 |
| 5 | Baseline LLM | 1.0000 | 0.4878 |
| 5 | MECR-RAG | 0.7000 | 0.6087 |
| 5 | Guideline-only | 0.9000 | 0.4500 |
| 5 | Case-only | 0.7000 | 0.5833 |

**Table S10. Scaling effects of retrieval database size on MECR-RAG performance (five runs per configuration).**

| Retrieval database size (cases) | QWK, mean (SD) | QWK, 95% CI | Accuracy, mean (SD) | Accuracy, 95% CI |
| --- | --- | --- | --- | --- |
| 1,000 | 0.861 (0.011) | 0.852–0.870 | 0.711 (0.013) | 0.695–0.727 |
| 2,000 | 0.883 (0.006) | 0.877–0.890 | 0.756 (0.016) | 0.739–0.778 |
| 3,000 | 0.902 (0.002) | 0.901–0.904 | 0.802 (0.008) | 0.796–0.801 |

Pairwise differences in performance

| Comparison (cases) | ΔQWK (mean difference) | ΔQWK, 95% CI | *P* value (QWK) | ΔAccuracy (mean difference) | ΔAccuracy, 95% CI | *P* value (accuracy) |
| --- | --- | --- | --- | --- | --- | --- |
| 1,000 vs 2,000 | +0.019 | 0.010–0.029 | *P*<.001 | +0.045 | 0.027–0.063 | *P*<.001 |
| 2,000 vs 3,000 | +0.021 | 0.017–0.026 | *P*<.001 | +0.046 | 0.030–0.062 | *P*<.001 |

**Textboxes (in Order of Appearance)**

**Textbox S1. Rationale for cross‑year retrieval design and temporal drift assessment**

To assess the model’s temporal generalisability and retrieval resilience, we evaluated 2023 test cases using a 2024 retrieval database rather than a contemporaneous corpus. While this does not reflect typical deployment—where new cases would query a database containing recent or cumulative historical cases—it served to stress‑test robustness under temporal drift. The triage guideline version remained unchanged between years, isolating the impact of variation in documentation and case distribution.

Seasonal patterns such as infectious surges and shifting casemix can bias both test and retrieval content if limited to the same period. In addition, nursing staff and documentation habits tend to be relatively stable within a given year; using the same‑year data for both testing and retrieval risks overestimating performance by introducing hidden dependencies between how cases are documented and how they are later retrieved. By deliberately mismatching years, we reduce this potential circularity and provide a more conservative assessment of how well the retrieval‑augmented system generalises across evolving clinical contexts.

**Textbox S2. Detailed preprocessing and document transformation pipeline**

The preprocessing pipeline transformed raw clinical documentation into structured formats for retrieval‑augmented generation, processing two primary knowledge sources: historical emergency department cases and clinical triage guidelines.

**Clinical case processing**
Test cases and past historical cases were provided as individual PDF files containing complete emergency department documentation. A four‑stage automated pipeline extracted text using *pdfplumber*, implemented anonymisation by removing patient identifiers from standardised document headers, extracted structured clinical information using regular expressions, and converted data to a hierarchical JSON format with sections for demographics, clinical presentation, vital signs, and case disposition.

**Guideline processing**
The latest version of the Hong Kong Accident and Emergency Triage Guidelines (HKAETG; version 6, last revision in 2022) was converted from the institutional DOCX document to a machine‑readable format through a DOCX→Markdown→JSON transformation pipeline. This process preserved the hierarchical content structure and decision matrices while enabling section‑based retrieval. LangChain’s *MarkdownHeaderTextSplitter* was used to segment the guideline into discrete sections stored with separate titles and content for targeted access during retrieval.

**Summarisation preprocessing**
Both clinical cases and triage guideline sections underwent LLM‑based summarisation before vector embedding to optimise retrieval effectiveness. Summaries were designed to capture key triage‑relevant elements (eg, demographics, presenting complaint, abnormal vital signs, and salient clinical context) while remaining concise enough for efficient semantic search. Detailed summarisation prompts, consistency protocols, and quality‑assurance procedures are described in the Methods section on indexing and database construction and reproduced in the Multimedia Appendix describing the indexing prompts.

**Textbox S3.** Confidence interval estimation and handling of LLM stochasticity

To account for the inherent variability in outputs generated by probabilistic large language models, all primary comparisons involving overall performance metrics (eg, QWK and accuracy) between the MECR‑RAG system and the baseline prompt‑only LLM were repeated five times under identical conditions. Each run used the same 236‑case test set, prompts, retrieval databases, and API settings (DeepSeek‑V3), with temperature set to 0 to reduce sampling variance while preserving reproducible stochastic behaviour.

For these repeated‑run analyses, we summarised performance using the mean and 95% confidence intervals (CIs) across the five runs. CIs were calculated using the t distribution applied to the sample of run‑level metric values, thereby capturing between‑run variability without inflating the effective sample size at the case level.

Some analyses—which required per‑case interpretability or would otherwise risk pseudo‑replication—were conducted on a single representative run (the median‑performing prediction set across the five runs). This applied, for example, to post hoc triage‑group analyses (Immediate/Urgent/Non‑Urgent), per‑category performance, and outcome‑based binary constructs. For these single‑run analyses, 95% CIs were estimated using non‑parametric bootstrapping with 1000 case‑level resampling iterations, drawing with replacement from the 236 test cases. For each bootstrap sample, we recomputed the metric of interest (eg, accuracy, sensitivity, specificity), and empirical percentile CIs were then derived from the bootstrap distribution. The CI method applied to each reported metric is explicitly noted in the Results section.

**Textbox S4.** Binary outcome constructs (“lenses”) for severity

We prespecified four binary outcome constructs (“lenses”) combining the R1–R3 tiers and the disposition‑safety composite (DSC_any) to examine robustness across different definitions of high severity:

1. **Classic lens (MTS‑style)**
   - Outcome+ = R1.
   - Outcome– = R2 or R3.
   - Test+ = triage Categories 1–2 (Immediate).
   - Test– = triage Categories 3–5.
2. **Operational lens (queue‑safety focus)**
   - Outcome+ = R1 or R2.
   - Outcome– = R3.
   - Test+ = triage Categories 1–3 (Immediate or Urgent).
   - Test– = triage Categories 4–5 (Non‑Urgent).
3. **Augmented classic lens (R1 or DSC)**
   - Outcome+ = R1 or DSC_any.
   - Outcome– = R2 or R3 without DSC_any.
   - Test+ = triage Categories 1–2.
   - Test– = triage Categories 3–5.
4. **Augmented operational lens (R1/R2/DSC)**
   - Outcome+ = R1 or R2 or DSC_any.
   - Outcome– = R3 without DSC_any.
   - Test+ = triage Categories 1–3.
   - Test– = triage Categories 4–5.

These lenses were chosen to reflect (a) traditional triage validation approaches focused on ICU admission and death (classic lens), (b) operational separation between patients who should not wait in the lowest‑priority queue and those who can safely do so (operational lens), and (c) augmented constructs that incorporate sentinel deterioration events (ICU transfer, early readmission, and short‑term mortality) into the definition of “true” high acuity (augmented lenses).

**Textbox S5.** Statistical methods for outcome‑based validity and harm index

For each binary lens and each triage method (nurse triage, baseline LLM, and MECR‑RAG), we constructed 2×2 tables cross‑classifying Test+ versus Outcome+. From these tables we calculated:

- **Sensitivity** = TP / (TP + FN).
- **Specificity** = TN / (TN + FP).
- **Positive likelihood ratio (LR+)** = Sensitivity / (1 − Specificity).
- **Negative likelihood ratio (LR–)** = (1 − Sensitivity) / Specificity.
- **Diagnostic odds ratio (DOR)** = (TP/FN) / (FP/TN).

Exact 95% confidence intervals for sensitivity and specificity were obtained using the Clopper–Pearson method. DOR CIs were calculated on the log scale using standard asymptotic formulas and then exponentiated back to the original scale. Given the modest number of R1 events and DSC_any cases, these estimates should be interpreted with particular caution.

To compare models on the same patients, we used continuity‑corrected paired McNemar tests separately within Outcome+ (for sensitivity differences) and Outcome– (for specificity differences). These tests evaluated whether discordant classifications (eg, MECR‑RAG correct and nurse incorrect vs nurse correct and MECR‑RAG incorrect) occurred more often in one direction than the other.

As complementary, clinically interpretable summaries, we report:

1. The proportion of R1 cases assigned to Immediate (Categories 1–2).
2. The proportion of R2 cases assigned to Non‑Urgent (Categories 4–5).
3. A weighted harm index per 100 patients.

The **weighted harm index** assigns higher penalties to more dangerous errors:

- R1 assigned to Non‑Urgent (Categories 4–5): weight = 5.
- R2 assigned to Non‑Urgent: weight = 2.
- R3 assigned to Immediate or Urgent (Categories 1–3): weight = 1.

For each case and each triage method, we computed a harm score (0 if correctly classified or misclassified within the same urgency band, or the relevant weight if misclassified in the above ways). The harm index per 100 patients was then calculated as:

$$\text{Harm index}=100\times\frac{\sum_{i=1}^{N} \text{harm}_{i}}{N}$$

where $N$is the number of cases with outcome labels. Paired sign tests were used to compare harm scores between methods at the individual‑case level (eg, MECR‑RAG vs baseline LLM, MECR‑RAG vs nurse triage).

**Textbox S6. Supplementary statistical and comparative analyses**

A series of additional analyses were conducted to characterise model performance across configurations, database sizes, and patient subgroups.

**Repeated evaluations and scaling analyses**

To examine the impact of retrieval database size, we evaluated MECR‑RAG with databases of 1000, 2000, and 3000 past triage cases. For each condition, the 236‑case test set was evaluated in five independent runs with identical prompts and API settings. Databases of 1000 and 2000 cases were constructed by random sampling from the original 3000‑case retrieval pool while preserving the overall triage category distribution. Mean QWK and accuracy with 95% CIs were computed across runs, and differences between database sizes were assessed using bootstrap resampling of the run‑level metrics (1000 resamples).

**Exploratory cross‑model generalisability**

To explore whether the retrieval framework generalised beyond DeepSeek‑V3, we implemented the same triage prediction pipeline (prompts, test set, and 3000‑case database) using Claude 3.7 and GPT‑4o. Each model was evaluated once under two configurations: baseline (no retrieval) and MECR‑RAG (dual‑source retrieval). These analyses were descriptive, intended to assess cross‑model robustness rather than formally compare commercial models.

**Subgroup analyses**

We conducted exploratory subgroup analyses stratified by sex (male, female) and age group (0–17, 18–64, ≥65 years) for the MECR‑RAG system. For each subgroup, QWK and accuracy were calculated across five runs and summarised as mean values with 95% bootstrap CIs (1000 resamples). Subgroup sizes were determined by the natural distribution of the test set and were not balanced or powered for formal hypothesis testing; accordingly, no formal statistical comparisons between subgroups were performed.

**Statistical tests and software**

All analyses were performed in R (version 4.5.0). Inter‑rater agreement and kappa statistics were calculated using the irr and psych packages. Classification performance metrics (precision, recall, F1, confusion matrices) were obtained using caret and yardstick. Non‑parametric CIs were estimated using the boot package. Between‑model comparisons used:

- McNemar’s test (function mcnemar.test in stats) for paired binary outcomes.
- Cochran’s Q test (function CochranQTest in DescTools) for >2 related proportions.
- Friedman tests (function friedman.test in stats) with Nemenyi post hoc comparisons for repeated‑run rank comparisons across model variants.

Visualisations were generated using ggplot2 and refined for publication using Adobe Illustrator and Adobe Photoshop. Large language models (eg, GPT‑4o and Claude 3.7) were used to assist with code drafting, debugging, and interpretation of diagnostic outputs; all analyses and scripts were checked by the investigators.

**Figures (in Order of Appearance)**

**
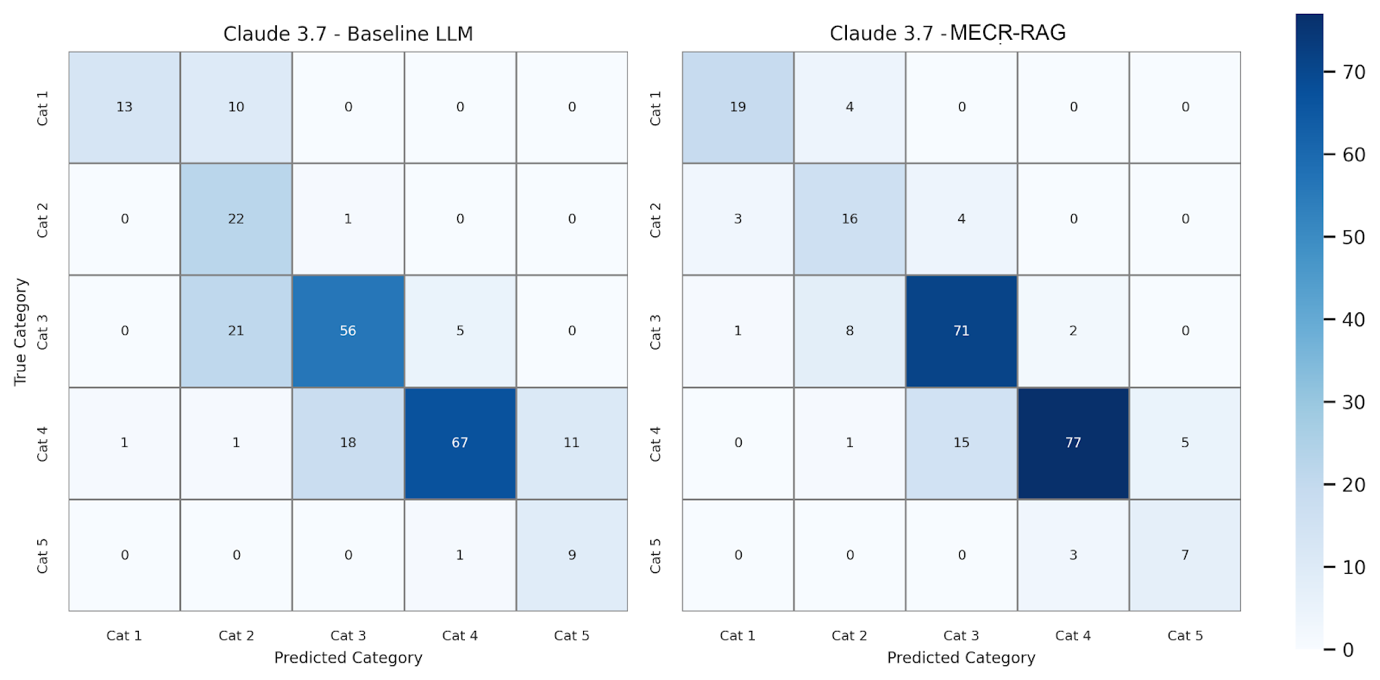
**

**Figure S1. Confusion matrices for Claude 3.7 triage predictions under baseline and retrieval‑augmented configurations.** The left panel shows five‑level triage category predictions from the baseline prompt‑only large language model (LLM) configuration, and the right panel shows predictions from the Multi‑Evidence Clinical Reasoning Retrieval‑Augmented Generation (MECR‑RAG) system, both evaluated on the same 236‑case emergency department test set. Rows represent expert consensus true triage categories (Categories 1–5), and columns represent model‑predicted categories. Cell values denote case counts, and shading intensity is proportional to frequency on a shared color scale, enabling direct comparison between configurations. The MECR‑RAG configuration shows greater concentration of cases along the main diagonal and fewer off‑diagonal errors, consistent with improved classification consistency.


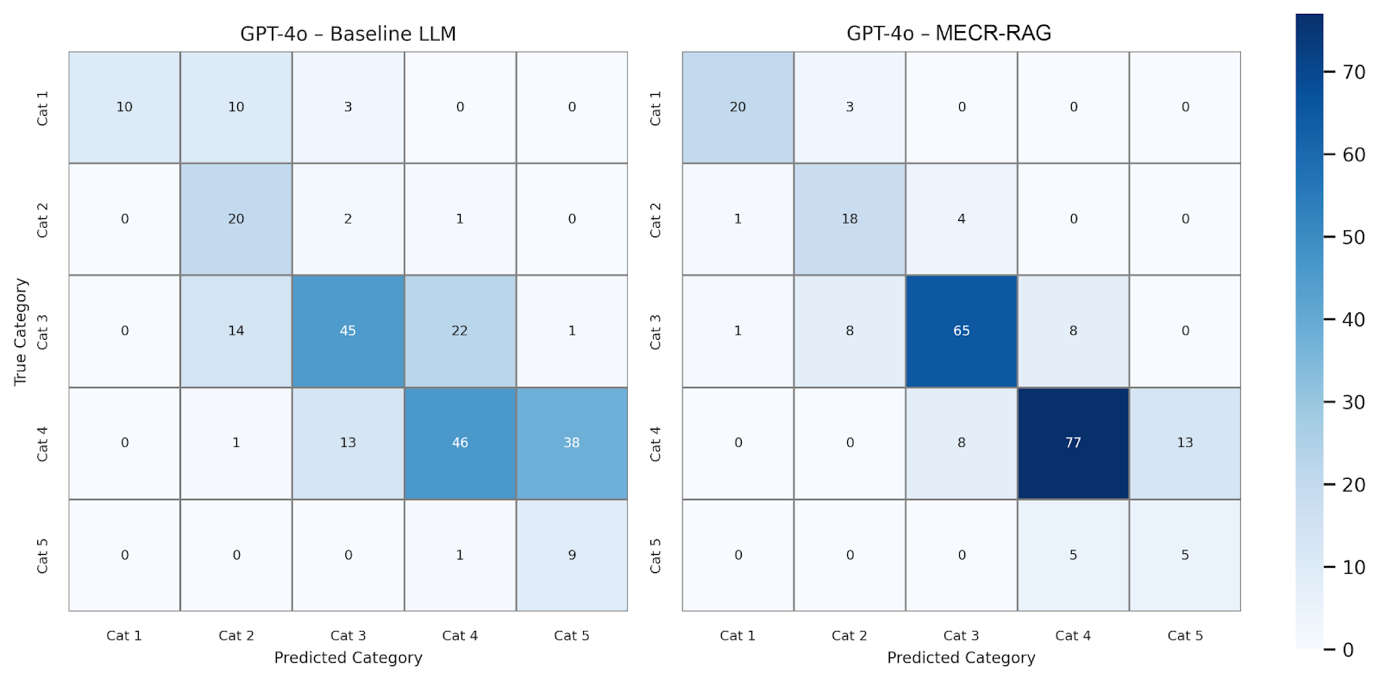


**Figure S2. Confusion matrices for GPT‑4o triage category predictions under baseline and retrieval‑augmented configurations.** The left panel shows five‑level triage category predictions from the baseline prompt‑only large language model (LLM) configuration, and the right panel shows predictions from the Multi‑Evidence Clinical Reasoning Retrieval‑Augmented Generation (MECR‑RAG) system, both evaluated on the same 236‑case emergency department test set. Rows represent expert consensus true triage categories (Categories 1–5), and columns represent model‑predicted categories. Cell values denote case counts, and shading intensity is proportional to frequency on a shared color scale, enabling direct comparison between configurations. The MECR‑RAG configuration shows greater concentration of cases along the main diagonal and fewer off‑diagonal errors, suggesting improved agreement with expert triage assignments. These results are based on a single inference run and provide an exploratory assessment of generalizability across large language model architectures.

**Sections A-D (in order of appearance)**

**Section A – Detailed LLM and RAG system implementation**

#### A1. Model architecture and technical implementation

Three state‑of‑the‑art large language models were evaluated in the retrieval‑augmented generation pipeline to assess cross‑model generalisability and performance consistency. DeepSeek‑V3‑0324 was accessed via the DeepSeek API using LangChain's ChatOpenAI interface; GPT‑4o model version 2024‑11‑20 was accessed through Azure OpenAI services using the AzureChatOpenAI interface with Azure deployment endpoints; and Claude‑3.7‑sonnet‑20250219 was accessed via Anthropic's API using LangChain's ChatAnthropic interface. All models utilised temperature=0 for deterministic generation, with the same LLM model processing all pipeline stages—preprocessing, retrieval, and generation—within each experimental condition to ensure fair comparison.

Vector embeddings for semantic retrieval utilised OpenAI's text‑embedding‑3‑small model version 1 accessed through Azure OpenAI services, maintaining consistent embedding generation across all experimental configurations.

The RAG system was implemented in Python (version 3.10.16) using LangChain for orchestrating language model interactions and document processing workflows. Vector databases were created and managed using Chroma for efficient semantic retrieval. Document processing utilised unstructured and python‑docx for medical document parsing and DOCX conversion, and pdfplumber for PDF text extraction. Data manipulation and analysis were performed using pandas and numpy, while environment management was handled through python‑dotenv. The modular pipeline architecture was implemented using LangGraph for state management and workflow orchestration across preprocessing, retrieval, and generation components.

This study utilised existing, pre‑trained commercial large language models without additional fine‑tuning, alignment modifications, instruction tuning, or architectural changes. The models were accessed via their respective APIs in their original, commercially available states, retaining their existing instruction‑following capabilities and safety alignments as implemented by the original developers. No custom model development, training, or alignment strategies were performed as part of this research.

All experiments were conducted on a local workstation equipped with an AMD Ryzen 9 7940HS processor (4.00 GHz) and 32 GB of RAM. As model inference was performed via commercial LLM APIs, no GPU acceleration was required. Replication of the pipeline can be achieved using any standard desktop computer with internet connectivity. The implementation used ChromaDB for vector storage and retrieval, LangChain for orchestration of the language model prompts and outputs, and standard Python scientific computing libraries for data processing and evaluation.

The average total processing time per case for the MECR‑RAG system, using DeepSeek‑V3 and a retrieval database of 3,000 triage cases, was 55.7 seconds (range 35.5–99.0 seconds). A breakdown of processing time showed average durations of 5.3 seconds for preprocessing, 3.0 seconds for guideline retrieval, 5.7 seconds for specialty prediction, 2.1 seconds for past‑case retrieval, and 39.6 seconds for final prediction generation. The retrieval of guidelines and past cases remained highly efficient even at this scale, and further expansion of the reference case database in future implementations is not expected to substantially increase total processing time.

#### A2. MECR‑RAG system architecture

The MECR‑RAG system implements a node‑based processing framework using LangGraph for workflow orchestration and LangChain for standardised LLM interactions. The system processes new emergency cases through five sequential stages: (1) clinical case summarisation for optimised retrieval, (2) agentic guideline section selection, (3) specialty prediction for metadata filtering, (4) hybrid past‑case retrieval combining metadata and vector similarity, and (5) multi‑evidence reasoning for final triage assessment. A GraphState class maintains all intermediate processing results, enabling complete audit trails and deterministic execution paths. The modular node architecture supports systematic ablation studies by enabling selective component inclusion while maintaining consistent underlying implementations.

#### A3. Indexing and database construction

Historical emergency cases underwent LLM‑based summarisation before vector embedding to optimise retrieval effectiveness. Drawing from Anthropic's contextual retrieval methodology, each case received a concise clinical summary emphasising demographics, presenting complaints, abnormal vital signs, and clinical context. The summarisation employed structured prompt engineering with explicit vital‑sign abnormality thresholds and medical terminology normalisation guidelines. The same summarisation prompt processed both historical database cases and new incoming cases, ensuring structural and semantic alignment in the embedding space and eliminating the need for query transformation techniques.

ChromaDB stored case summaries with cosine similarity metrics optimised for clinical semantic search. The system integrated metadata alongside vector embeddings, enabling hybrid search approaches combining semantic similarity with structured clinical attributes including age groups, attending specialties, and triage categories. The system supported configurable database sizes (1,000, 2,000, and 3,000 cases) with proportional sampling across triage categories to evaluate knowledge‑base size effects while maintaining statistical validity through randomisation and balanced representation.

Clinical guidelines underwent LLM‑based summarisation optimised for relevance assessment during retrieval. Each guideline section received a structured summary beginning with "Consult for patients with..." and describing the spectrum from minimum threshold conditions to severe presentations, maintaining concise 20–30 word summaries using medical terminology.

#### A4. Retrieval system design

The retrieval system implements a dual‑source approach combining agentic guideline selection with hybrid metadata–vector similarity search for past cases, addressing the distinct retrieval requirements of authoritative protocols versus experiential case patterns.

For guidelines, the system employs LLM‑based intelligent selection rather than pure similarity‑based matching. The model receives complete guideline metadata (section titles and summaries) alongside the clinical case summary and applies clinical reasoning to select a maximum of two most critical sections, prioritising life‑threatening conditions and documented abnormal vital signs meeting specific thresholds.

Past‑case retrieval implements a four‑stage hybrid approach. First, agentic specialty prediction addresses information asymmetry by predicting both primary and secondary attending specialties from triage‑available information only. Second, metadata filtering constructs filters combining demographic criteria (adult/pediatric classification) with predicted specialty mappings to dataset values. Third, iterative specialty filtering creates separate filter combinations for each specialty variant to maximise case discovery while maintaining clinical relevance. Fourth, constrained vector similarity search performs cosine similarity calculations only within the clinically relevant subset identified through metadata filtering.

Vector similarity search employs a similarity score threshold of 0.7 (converted from ChromaDB distance scores as similarity = 1 − distance) combined with top‑k retrieval (k=5) to ensure high‑quality, semantically relevant cases. The system includes comprehensive fallback mechanisms expanding to all available cases when specialty‑based filtering yields insufficient results. All vector operations utilise the same text‑embedding‑3‑small model, maintaining consistent embedding generation across both database construction and query processing phases to ensure reliable similarity calculations.

#### A5. Multi‑Evidence Clinical Reasoning (MECR) prompting

The generation framework implements the MECR prompting methodology, extending traditional chain‑of‑thought reasoning specifically for clinical decision‑support contexts where multiple evidence sources require systematic integration. Building upon foundational chain‑of‑thought prompting, the MECR framework implements a structured three‑tier evidence‑integration approach: (1) clinical risk assessment aligned with standardised triage definitions, (2) evidence‑based guideline integration applying condition‑specific recommendations, and (3) historical case pattern analysis capturing demographic and contextual factors influencing real‑world decisions beyond formal protocols.

The MECR framework implements an ensemble‑based decision mechanism where each evidence source provides an independent triage category assessment. Clinical risk evaluation yields an initial category based on acuity definitions, guideline‑based assessment provides condition‑specific recommendations, and historical case analysis offers real‑world adjustment factors. The final decision synthesises these multiple perspectives through structured comparison and justification, leveraging the concept that different knowledge sources possess complementary strengths rather than employing multiple LLM architectures.

The generation prompts embed comprehensive triage category definitions in tabular format, ensuring all decisions ground in standardised clinical protocols. The MECR framework enforces rigid stepwise analytical structure mirroring established clinical reasoning patterns while mapping each step to specific knowledge sources. The prompts explicitly instruct systematic analysis of all provided evidence sources while maintaining analytical rigor.

Outputs explicitly document end‑to‑end reasoning processes, including category assignments from each reasoning step, confidence levels (High/Medium/Low), and detailed rationale identifying which evidence sources influenced final decisions. The complete pipeline maintains full traceability from input case documentation through knowledge retrieval (guideline sections and past cases with similarity scores) to final MECR analysis, enabling healthcare professionals to audit and validate each component of the decision‑making process.


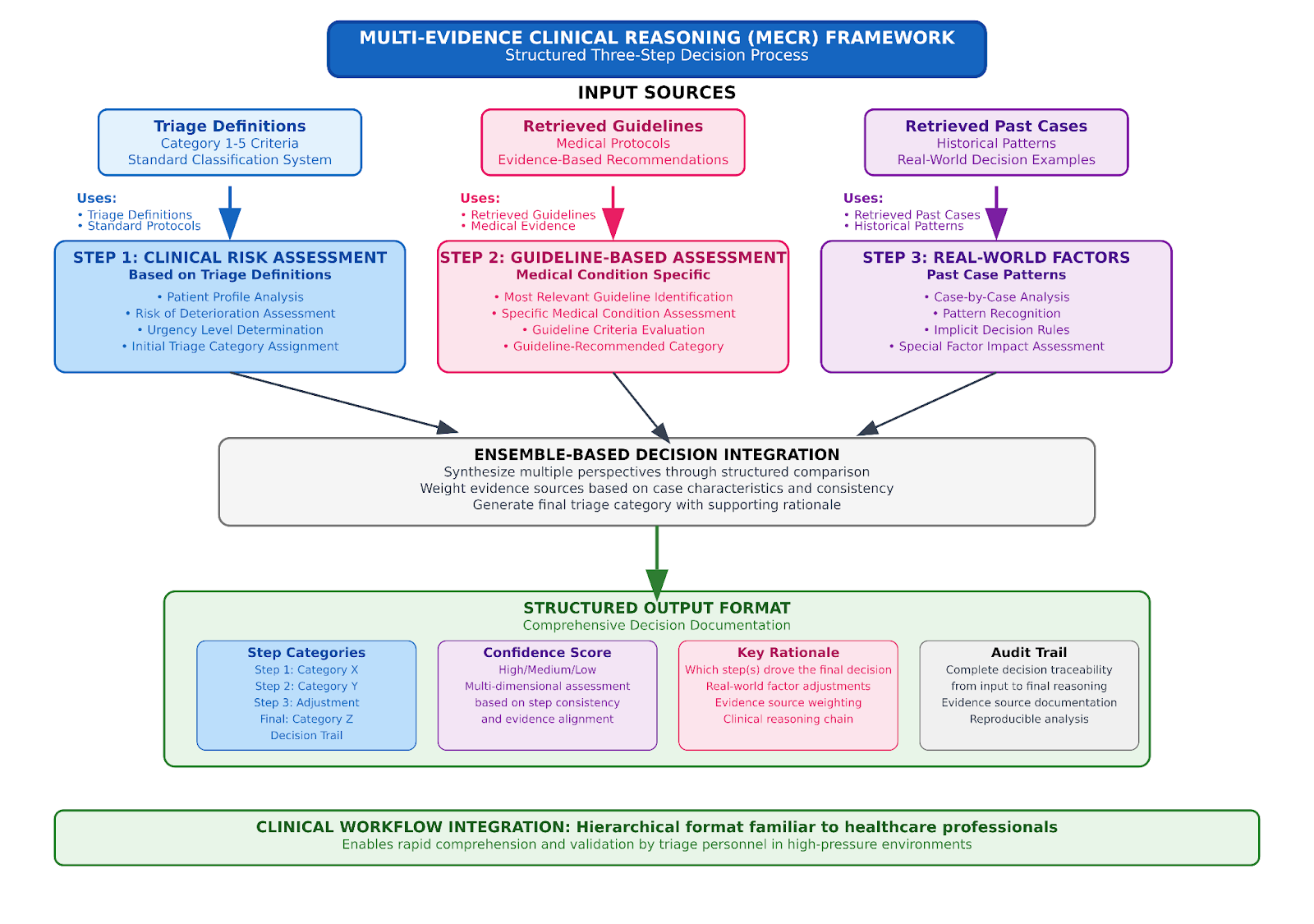


**Complete MECR Prompt Structure Demonstration**. Multi-Evidence Clinical Reasoning (MECR) framework implementing structured three-tier evidence integration for emergency triage decision-making. The framework systematically combines (1) clinical risk assessment based on standardized triage definitions, (2) evidence-based guideline integration applying condition-specific medical protocols, and (3) historical case pattern analysis incorporating demographic and contextual factors influencing real-world clinical decisions.

#### A6. Ablation study configurations and prompts

The ablation study systematically evaluated four experimental configurations to isolate the contribution of individual retrieval components within the MECR‑RAG architecture. The three reduced configurations maintained identical underlying node implementations with selective component inclusion compared to the complete system.

- **Baseline LLM configuration:** A prompt‑only large language model without any retrieval augmentation. The generation prompt provided comprehensive triage category definitions in tabular format with instructions for clinical assessment and rationale provision, establishing baseline performance without external knowledge integration.
- **Guideline‑only RAG configuration:** Augmented the baseline with agentic guideline selection and content retrieval while excluding past‑case retrieval. The generation framework implemented a two‑step reasoning approach: clinical risk assessment based on triage definitions and guideline‑based assessment applying specific medical protocols to documented conditions.
- **Case‑only RAG configuration:** Included the complete specialty‑prediction and hybrid metadata–vector case‑retrieval pipeline while excluding guideline components. The generation framework employed a two‑step approach: clinical risk assessment and real‑world factors analysis examining retrieved cases for demographic, social, and contextual factors influencing triage decisions beyond formal guidelines.
- **MECR‑RAG configuration:** Combined both guideline and case retrieval in a dual‑source architecture designed to emulate how triage experts integrate protocols with prior experience.

All configurations shared identical preprocessing nodes, LLM model assignments, and evaluation frameworks. The modular LangGraph architecture enabled systematic component removal without reimplementation, ensuring that performance differences reflected retrieval‑augmentation effects rather than implementation variations.


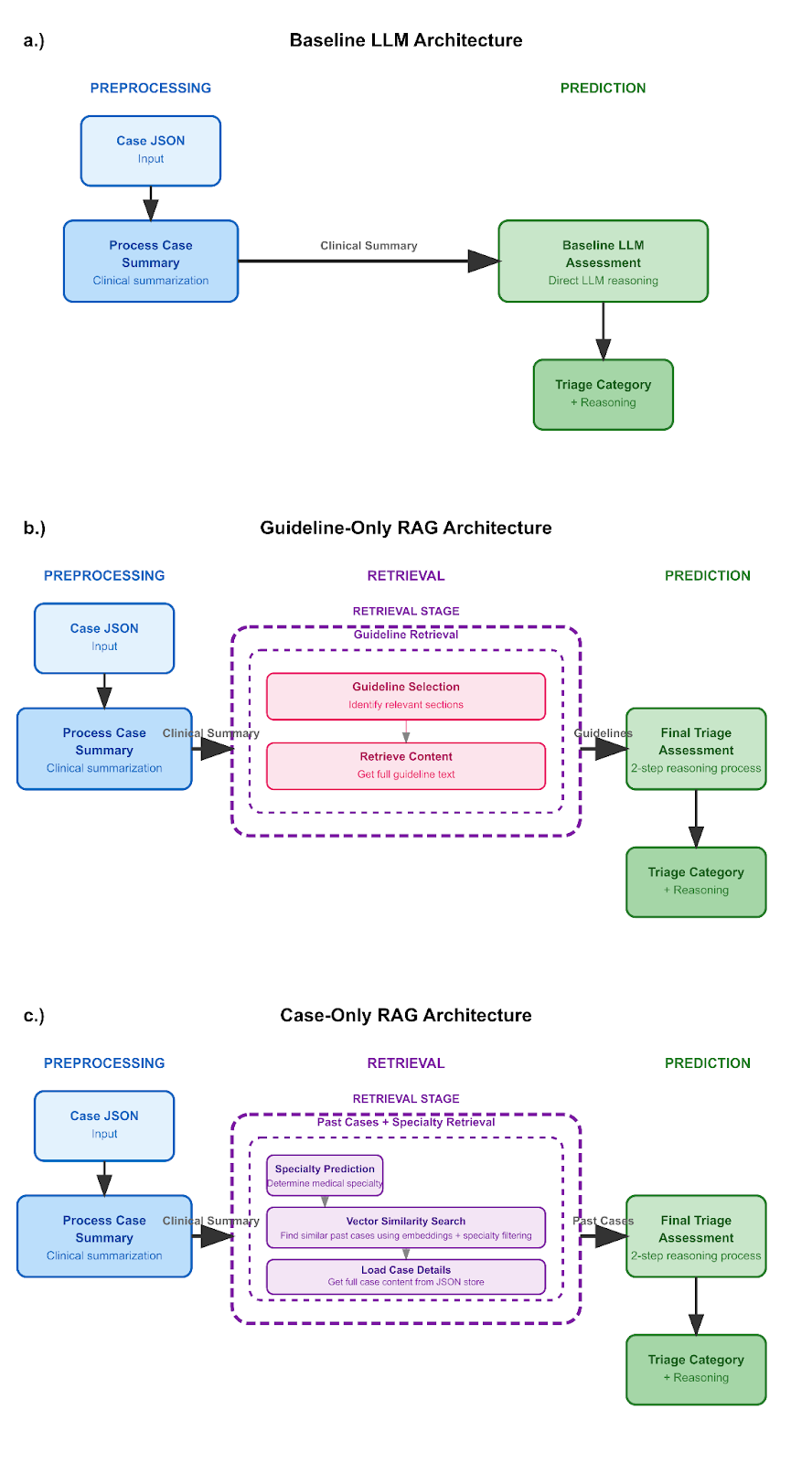


**Ablation study configurations**
Systematic ablation study configurations showing three reduced MECR‑RAG variants compared to the complete system: (a) baseline LLM configuration with case summarisation and direct triage prediction only; (b) guideline‑only RAG configuration augmenting baseline with agentic guideline retrieval; and (c) case‑only RAG configuration implementing specialty prediction and hybrid past‑case retrieval while excluding guidelines.

**Section B.** **Outcome‑based sensitivity analyses and disposition‑safety composite**

**Disposition‑safety composite (DSC) events**

Among 226 encounters with complete outcome data, 19 (8.4%) met the DSC_any criteria. These included unplanned ICU transfer within 72 hours of ward admission in 3 of 19 cases (15.8%), ED revisit within 72 hours resulting in unplanned admission in 1 of 19 (5.3%), and all‑cause death within 7 days in 15 of 19 (78.9%). No 72‑hour revisit with ICU admission occurred in this cohort. DSC_any was coded as positive if ≥1 component was present.

**Classic Manchester‑style lens (Outcome+ = R1 only)**

Under a classic Manchester Triage System–style lens (Outcome+ = R1 only; Outcome– = R2 or R3; Test+ = Categories 1–2 vs 3–5), all three systems achieved perfect sensitivity for R1 cases (13/13 each). Specificity for lower‑severity encounters differed: 84.0% (179/213) for nurses, 65.7% (140/213) for the baseline LLM, and 79.8% (170/213) for MECR‑RAG. The corresponding positive likelihood ratios (LR+) were higher for MECR‑RAG (4.95) than for the baseline model (2.92), with nurses achieving the highest LR+ because of their slightly higher specificity. These findings suggest that, when judged solely on R1 outcomes, MECR‑RAG narrows the gap between nurse performance and the baseline model by reducing false positives in lower‑severity cases.

**Augmented classic lens (Outcome+ = R1 or DSC_any)**

When the disposition‑safety composite (DSC_any) was added to define augmented outcomes (Outcome+ = R1 or DSC_any; Outcome– = all other non‑LWBS encounters; Test+ = Categories 1–2), there were 27 of 226 high‑severity cases (R1 or DSC_any). MECR‑RAG achieved a sensitivity of 96.3% (26/27) and specificity of 84.9% (169/199), with a diagnostic odds ratio (DOR) of 146.5 (95% CI 19.1–1120.5). In comparison, the baseline LLM yielded a sensitivity of 92.6% (25/27) and specificity of 79.9% (159/199), corresponding to a DOR of 60.2 (95% CI 8.0–454.1). Nurse triage achieved sensitivity 77.8% (21/27) and specificity 78.4% (156/199), with a DOR of 23.3 (95% CI 8.6–63.1). Although confidence intervals were wide and overlapping, MECR‑RAG consistently demonstrated the highest point estimates for both discrimination and overall trade‑off between sensitivity and specificity.

**Augmented operational lens (Outcome+ = R1 or R2 or DSC_any)**

In the augmented operational lens (Outcome+ = R1 or R2 or DSC_any; Outcome– = R3 without DSC_any; Test+ = Categories 1–3 vs 4–5), MECR‑RAG again showed the most favourable performance. Among 226 encounters, 131 met the augmented high‑severity definition. MECR‑RAG achieved sensitivity 95.4% (125/131) and specificity 81.1% (77/95), yielding a DOR of 89.1 (95% CI 33.9–234.3). By comparison, nurses achieved sensitivity 90.1% (118/131) and specificity 79.0% (75/95), with a DOR of 41.6 (95% CI 19.2–90.6); the baseline LLM showed sensitivity 93.1% (122/131) and specificity 68.4% (65/95), with a DOR of 29.4 (95% CI 13.2–65.6). In this lens, MECR‑RAG thus provided the highest discrimination between high‑ and low‑severity courses while maintaining a safety‑oriented balance between under‑ and overtriage.

Taken together, these sensitivity analyses across classic and augmented outcome lenses indicate that point estimates consistently favor MECR‑RAG over both nurse triage and the baseline LLM across multiple outcome‑based constructs of severity, although the relatively small number of R1 and DSC_any events and the wide, overlapping confidence intervals mean these findings should be interpreted cautiously.

**Section C Subgroup Analysis by Age and Sex**

To evaluate the demographic consistency and generalisability of the MECR-RAG system, we performed an exploratory subgroup analysis stratified by sex and age. This analysis was descriptive in nature and not statistically powered for formal inferential comparisons. Performance metrics (QWK and accuracy) were calculated for each subgroup based on five stochastic inference runs.

Among female patients (n = 105), the MECR-RAG achieved a mean QWK of 0.890 (95% CI: 0.880 to 0.900) and accuracy of 0.815 (95% CI: 0.793 to 0.838). In male patients (n = 131), mean QWK was 0.907 (95% CI: 0.900 to 0.914) and accuracy was 0.791 (95% CI: 0.774 to 0.808), with overlapping confidence intervals observed across sexes.

When stratified by age, adults aged 18–64 years (n = 105) had the highest model agreement with reference labels, with QWK of 0.917 (95% CI: 0.915 to 0.919) and accuracy of 0.843 (95% CI: 0.831 to 0.856). In older adults aged ≥65 years (n = 103), QWK was 0.878 (95% CI: 0.870 to 0.886) and accuracy 0.766 (95% CI: 0.750 to 0.781). For pediatric patients aged 0–17 years (n = 28), QWK was 0.716 (95% CI: 0.658 to 0.777) and accuracy was 0.786 (95% CI: 0.721 to 0.850). The above findings are summarised in the following table:

Although small differences were observed across subgroups, all estimates remained within a high-performing range. Wider confidence intervals in the pediatric group likely reflect the smaller sample size, limiting interpretability in this subgroup. Overall, these findings suggest no obvious degradation of MECR‑RAG performance across sex and age subgroups within this cohort, although the small paediatric sample (n=28) and wider confidence intervals mean that generalisability to under‑represented groups remains uncertain and requires further validation.

**Section D. Exploratory Performance on adjudicated disagreement case**

An exploratory evaluation was conducted on cases considered triage edge cases—defined as those with disagreement between professional raters or between the original nurse-assigned category and the consensus label. To assess model performance in clinically ambiguous scenarios, we analyzed two subsets of cases where human disagreement was present. The first subset included 45 cases in which two professional raters assigned different triage categories, and the final consensus was determined by a third professional. The second subset consisted of 28 cases where the initial nurse triage category differed from the final consensus.

For these analyses, each case was run five times per model, and the most frequently predicted category was selected as the model’s final output. This majority-vote approach was used in lieu of selecting a single median run to improve stability in model predictions while preserving the one-case-one-label evaluation structure. This method mitigates random noise from probabilistic output generation and avoids pseudoreplication, thereby offering a more robust estimate of model behaviour in clinically ambiguous cases.

On rater disagreement cases, MECR-RAG achieved an accuracy of 84.4% (95% CI 73.3–93.3), outperforming the baseline LLM at 66.7% (95% CI 53.3–80.0), with a directional trend favoring MECR-RAG (McNemar P=.057). The model corrected prior human disagreement in 38 of 45 cases, compared to 30 for the baseline model, and exhibited fewer divergent errors (1 vs 7).

On cases involving disagreement between the initial nurse triage and the final consensus, MECR-RAG achieved an accuracy of 75.0% (95% CI 57.1–89.3), which was higher than the baseline LLM at 60.7% (95% CI 42.9–78.6), though the difference was not statistically significant (P=.343). MECR-RAG corrected the nurse’s triage in 21 of 28 cases, compared to 17 for the baseline model, and made fewer divergent errors (1 vs 3).

Together, these exploratory findings with small sample size illustrate that MECR‑RAG may resolve expert disagreement more often than the baseline LLM in this small sample, particularly by reducing novel or inconsistent errors.
